# An inducible Cre mouse for studying roles of the RPE in retinal physiology and disease

**DOI:** 10.1172/jci.insight.146604

**Published:** 2021-05-10

**Authors:** Elliot H. Choi, Susie Suh, David E. Einstein, Henri Leinonen, Zhiqian Dong, Sriganesh Ramachandra Rao, Steven J. Fliesler, Seth Blackshaw, Minzhong Yu, Neal S. Peachey, Krzysztof Palczewski, Philip D. Kiser

**Affiliations:** 1 Department of Ophthalmology, Gavin Herbert Eye Institute, University of California, Irvine, Irvine, California, USA.; 2 Department of Pharmacology, School of Medicine, Case Western Reserve University, Cleveland, Ohio, USA.; 3 Department of Physiology and Biophysics, School of Medicine, University of California, Irvine, Irvine, California, USA.; 4 Research Service, VA Long Beach Healthcare System, Long Beach, California, USA.; 5 Departments of Ophthalmology and Biochemistry, Jacobs School of Medicine and Biomedical Sciences and; 6 Neuroscience Graduate Program, University at Buffalo, The State University of New York, Buffalo, New York, USA.; 7 Research Service, VA Western New York Healthcare System, Buffalo, New York, USA.; 8 Department of Neuroscience, Johns Hopkins University School of Medicine, Baltimore, Maryland, USA.; 9 Department of Ophthalmic Research, Cole Eye Institute, Cleveland Clinic Foundation, Cleveland, Ohio, USA.; 10 Department of Ophthalmology, Cleveland Clinic Lerner College of Medicine of Case Western Reserve University, Cleveland, Ohio, USA.; 11 Research Service, Louis Stokes Cleveland VA Medical Center, Cleveland, Ohio, USA.; 12 Department of Chemistry, School of Physical Sciences, University of California, Irvine, Irvine, California, USA.

**Keywords:** Genetics, Ophthalmology, Mouse models, Retinopathy

## Abstract

The retinal pigment epithelium (RPE) provides vital metabolic support for retinal photoreceptor cells and is an important player in numerous retinal diseases. Gene manipulation in mice using the Cre-*LoxP* system is an invaluable tool for studying the genetic basis of these retinal diseases. However, existing RPE-targeted Cre mouse lines have critical limitations that restrict their reliability for studies of disease pathogenesis and treatment, including mosaic Cre expression, inducer-independent activity, off-target Cre expression, and intrinsic toxicity. Here, we report the generation and characterization of a knockin mouse line in which a P2A-CreER^T2^ coding sequence is fused with the native RPE-specific 65 kDa protein (*Rpe65*) gene for cotranslational expression of CreER^T2^. *Cre^+/–^* mice were able to recombine a stringent Cre reporter allele with more than 99% efficiency and absolute RPE specificity upon tamoxifen induction at both postnatal days (PD) 21 and 50. Tamoxifen-independent Cre activity was negligible at PD64. Moreover, tamoxifen-treated *Cre^+/–^* mice displayed no signs of structural or functional retinal pathology up to 4 months of age. Despite weak RPE65 expression from the knockin allele, visual cycle function was normal in *Cre^+/–^* mice. These data indicate that *Rpe65^CreERT2^* mice are well suited for studies of gene function and pathophysiology in the RPE.

## Graphical abstract

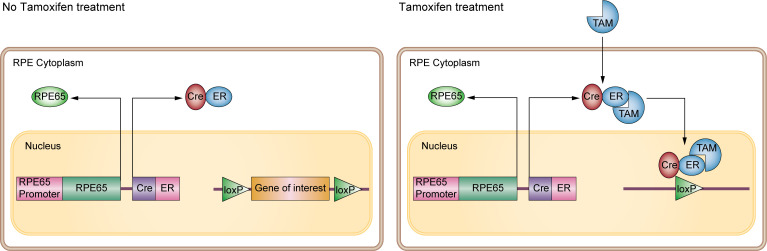

## Introduction

The retinal pigment epithelium (RPE) is a monolayer of cells that closely interacts with the photoreceptors of the retina and plays essential roles in the maintenance of visual function (1–3). The RPE critically supports the photochemistry of vision by supplying the visual chromophore, 11-*cis*-retinal, to rod and cone opsins, thereby generating functional visual pigments (4, 5). It also protects photoreceptors by forming the outer blood-retinal barrier, allowing selective transport of nutrients and waste products between the photoreceptor cells and the systemic circulation, and contributes to the maintenance of the photoreceptor outer segments (6) and the choroid (7). The basolateral membrane of the RPE faces Bruch’s membrane and is the site for the exchange of nutrients and waste products with the systemic circulation (8–10). The apical microvillar membranes of the RPE phagocytize the distal tips of the photoreceptor outer segments as part of a daily outer segment renewal process (11, 12). RPE dysfunction underlies a broad spectrum of retinal diseases, including age-related macular degeneration, retinitis pigmentosa, Sorsby fundus dystrophy, and fundus albipunctatus, among others (13–17). In each case, genetic factors are pivotal in the disease etiology and pathogenesis (18).

Mouse models with genetic alterations mimicking those of human retinopathies have proved invaluable tools for understanding the pathogenesis of and designing treatment strategies for retinal disorders (19, 20). Because many RPE-associated functional target genes are expressed in other cell types or play important roles during development, global knockout mice are often inappropriate or inadequate to address functional or pathogenic roles of genes specifically in the mature RPE. The Cre-*LoxP* recombinase system is one of the most effective and versatile tools available for conditional excision of a target DNA sequence (21), and there have been significant efforts to generate RPE-specific Cre mouse lines (Supplemental Table 1; supplemental material available online with this article; https://doi.org/10.1172/jci.insight.146604DS1). Noninducible RPE-targeted Cre mouse lines reported to date include the dopachrome tautomerase Cre (22), the tyrosinase related-protein-1 Cre (23), the melanoma-associated antigen recognized by T cells Cre (24), and the bestrophin 1 (*BEST1*) Cre (25) mouse lines. Inducible RPE-targeted Cre mice also have been generated and include the monocarboxylate transporter 3 CreER^T2^ (26), the tet-ON VMD2-Cre (27), and the tyrosinase CreER^T2^ (28). All of these Cre mouse lines suffer from various inadequacies that limit their usefulness, including mosaic patterns of Cre expression in the RPE, inducer-independent (i.e., “leaky”) Cre activity, off-target Cre expression, and, in the case of some constitutively active systems, Cre-mediated toxicity to the RPE (29, 30). Although a recently described *BEST1*-CreER^T2^ mouse line (31) showed improved recombination efficiency and RPE selectivity as compared with prior inducible lines, it still did not achieve complete and specific recombination within the RPE. Hence, a critical need remains for a reliable, nontoxic, RPE-specific Cre mouse line that can achieve quantitative recombination of floxed alleles in a temporally controlled manner. Such an animal model would significantly accelerate our understanding of RPE (patho)biology and assist in discovery of experimental therapies.

Toward this goal, we generated an inducible RPE-targeted Cre mouse line (*Rpe65^CreERT2^*) in which CreER^T2^ is bicistronically expressed with the RPE-specific 65 kDa protein (RPE65) via a P2A-CreER^T2^ knockin (KI) at the native *Rpe65* gene. Here, we report the characteristics of this mouse line, demonstrating its usefulness as a tool for studying gene function in a temporal fashion within the RPE.

## Results

### Generation of RPE65-P2A-CreER^T2^ KI mice.

To select an optimal gene for generation of an RPE-specific inducible Cre KI mouse, we analyzed mouse retina single-cell RNA-Seq data for transcripts with selective expression within the RPE. Our initial list included *Rpe65*, lecithin:retinol acyltransferase (*Lrat*), *Best1*, and bestrophin 2 (*Best2*) (32). Of these 4 genes, *Rpe65* exhibited the highest mRNA expression and had the added advantage of being RPE specific, as opposed to the other 3 genes, which are also expressed in nonretinal tissues, including the brain (*Best1* and *Best2*) and liver (*Lrat*) (33). Based on this analysis, *Rpe65* was chosen as the target gene for this study.

**Figure 1:**
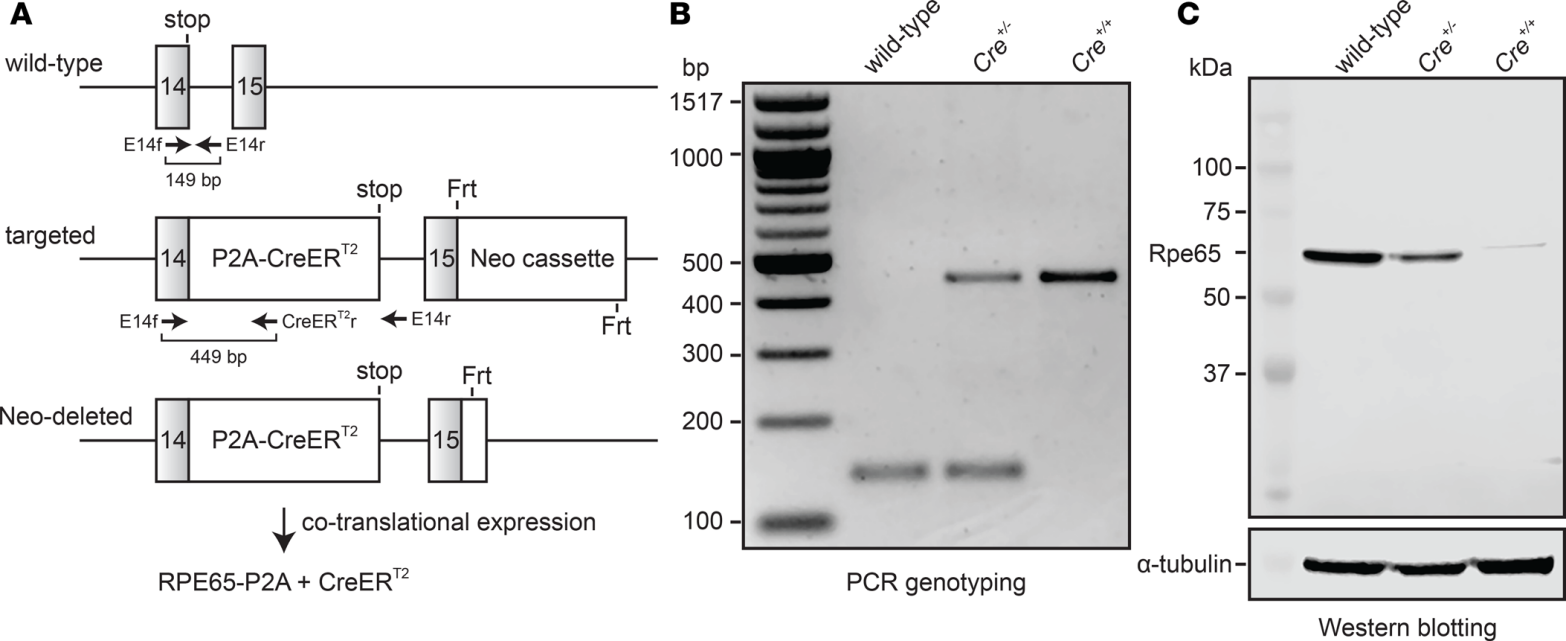
Generation, identification, and Rpe65 expression analysis of Rpe65^CreERT2^ mice. (**A**) KI strategy to introduce a P2A-CreER^T2^ coding sequence in-frame with the final coding exon (exon 14) of the *Rpe65* gene. Primer-binding sites and expected PCR product sizes are shown below the wild-type and targeted alleles. The neomycin cassette in the targeting vector was removed during expansion of the embryonic stem cell clone. FLP-recombinase was subsequently bred out by crossing with C57BL/6 mice. The KI allele allows cotranslational expression of RPE65-P2A and CreER^T2^ as 2 separate polypeptides. The modified RPE65 protein contains an additional G^534^SGATNFSLLKQAGDVEENPG^554^ polypeptide sequence at its C-terminus, while CreER^T2^ contains an additional Pro residue at its N-terminus. (**B**) Genotyping results from wild-type, *Cre^+/–^*, and *Cre^+/+^* animals. (**C**) Western blot analysis of RPE65 expression. After normalization to the α-tubulin loading controls, the RPE65 expression levels in *Cre^+/–^* and *Cre^+/+^* mice were estimated to be 59.7% and 1.1% of that for wild-type mice.

We generated a KI mouse line with a P2A-CreER^T2^ sequence fused in-frame after the last coding exon of the native *Rpe65* gene, which encodes the retinoid isomerase of the classical visual cycle (34) (Figure 1A). P2A is a porcine teschovirus-derived nucleotide sequence that causes high-efficiency ribosome skipping during translation (35, 36), thereby producing 2 separate proteins from a single transcript (Figure 1A). CreER^T2^ is an optimized Cre recombinase–estrogen receptor fusion protein that selectively undergoes nuclear translocation upon binding the exogenous agonist, 4-hydroxy-tamoxifen (4-OHT), allowing temporal control of Cre activity (37). We hypothesized that this KI approach, as opposed to introduction of an *Rpe65* promoter-driven CreER^T2^ transgene, would best ensure RPE cell specificity of CreER^T2^ expression and would avoid potential repeat-induced transgene silencing that may underlie the mosaicism observed for other RPE-targeted Cre lines (38, 39). These KI mice were crossed with both C57BL/6J mice (M450 RPE65 variant with lower enzymatic activity) and 129S1 mice (L450 RPE65 variant with normal enzymatic activity), and heterozygotes were then in-crossed to generate wild-type, heterozygous (*Cre^+/–^*), and homozygous (*Cre^+/+^*) mice for further breeding and analysis (Figure 1B). Both *Cre^+/–^* and *Cre^+/+^* mice were fully viable, reproduced normally, and had no gross phenotypic abnormalities.

We tested the impact of the KI sequence manipulation on RPE65 protein expression by performing Western blot analysis on isolated RPE lysates obtained from wild-type, *Cre^+/–^*, and *Cre^+/+^* mice, probing the blots with an in-house–generated monoclonal anti-RPE65 antibody (40). We observed that RPE65 expression in *Cre^+/–^* mice was approximately 60% of that in wild-type mice whereas expression in *Cre^+/+^* mice was much weaker (~1%) (Figure 1C). Additionally, the RPE65 band observed for *Cre^+/+^* mice migrated slightly slower than the wild-type protein. This is in agreement with the slightly higher apparent molecular weight (M_r_ ~2100 Da) of the RPE65-P2A fusion protein, which contains 21 non-native amino acid residues appended to the C-terminus of RPE65. These characteristics were consistent for mice with the 2 genetic backgrounds, indicating that expression of the modified RPE65 protein was not influenced by the variant type of the coexpressed wild-type RPE65. Quantitative PCR analysis revealed a small but nonsignificant reduction in *Rpe65* mRNA levels in *Cre^+/–^* and *Cre^+/+^* mice as compared with wild-type controls, indicating that the reduced protein expression does not result from a transcriptional deficit (Supplemental Figure 1).

### Assessment of LoxP recombination specificity and efficiency.

We assessed Cre-mediated recombination activity of the *Rpe65^CreERT2^* mice by crossing them with the B6.129(Cg)-Gt(ROSA)26Sor*^tm4(ACTB–tdTomato,–EGFP)Luo^*/J (mT/mG) Cre reporter mouse strain (41), which is known from lineage tracing studies to exhibit stringent recombination properties (42, 43). In this model, Cre recombinase activity at the reporter allele abolishes expression of a membrane-targeted tandem-dimer Tomato (mT) fusion protein with simultaneous activation of membrane-targeted green fluorescent protein (mG) expression via excision of the floxed mT-coding region. We tested recombination efficiency and selectivity in mice heterozygous for both the *Rpe65^CreERT2^* and the mT/mG reporter alleles at postnatal day (PD) 21 and PD50 by administering tamoxifen (a 4-OHT prodrug) by intraperitoneal (IP) injection for 5 consecutive days (1 mg on days 1–3 and 2 mg on days 4–5). Alternatively, tamoxifen was delivered in the chow diet (250 mg/kg of chow) for 3 weeks. Following completion of the induction regimen, mice were maintained under normal vivarium conditions for 2 more weeks to allow time for degradation of the mT protein that was produced prior to the induction treatment (41). Cre-mediated recombination was assessed via fluorescence microscopy in both RPE flatmounts and retina cryosections.

**Figure 2:**
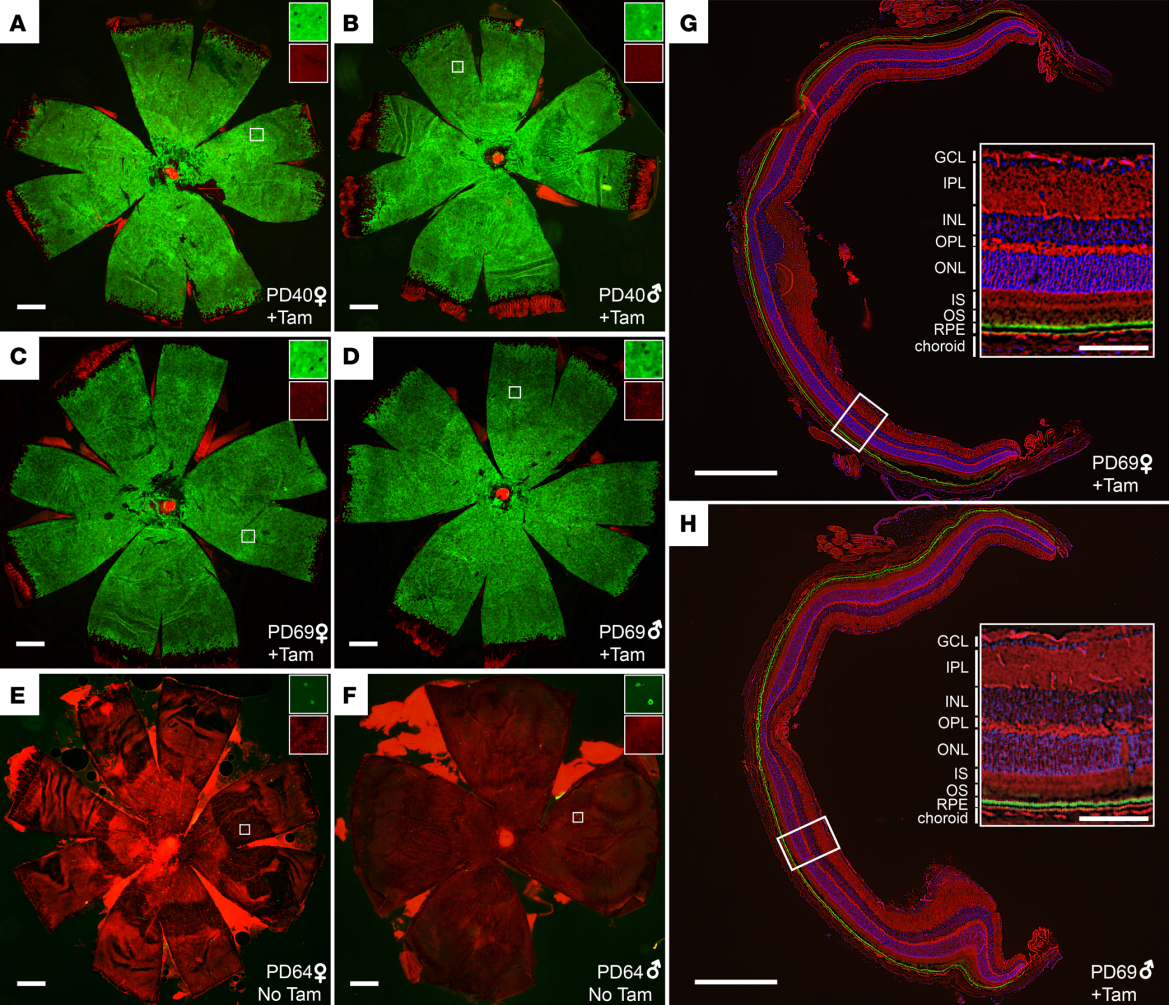
Cre recombinase activity in Rpe65^CreERT2^ mouse retina assessed with the mT/mG reporter. (**A** and **B**) RPE flatmounts from representative *Cre^+/–^* female and male mice, respectively, that were administered IP tamoxifen for 5 consecutive days starting on PD21 (*n* = 4). The flatmounts were obtained 2 weeks after completion of the tamoxifen regimen and were imaged with a fluorescence microscope. Green fluorescence indicates cells where Cre-mediated recombination has occurred, whereas red fluorescence indicates un-recombined cells. (**C** and **D**) RPE flatmounts from representative *Cre^+/–^* female and male mice that were administered IP tamoxifen for 5 consecutive days starting on PD50 (*n* = 7). The flatmounts were obtained 2 weeks after completion of the induction regimen. (**E** and **F**) show representative RPE flatmounts from *Cre^+/–^* female and male mice, respectively, that were not treated with tamoxifen (*n* = 6). The flatmounts were obtained on PD64. Scale bars indicate 500 μm. The insets in **A**–**F** show zoomed areas (marked by white squares, original magnification, 10×) with green and red channels separately displayed to demonstrate the single-cell resolution of the imaging method. (**G** and **H**) Retina cryosections from *Cre^+/–^* female and male mice, respectively, that were administered IP tamoxifen for 5 consecutive days starting on PD21 (*n* = 2). The cryosections were obtained 2 weeks after completion of the induction regimen. Insets show zoomed views of the areas marked with white rectangles with the retinal cell layers labeled. The green label represents Cre-mediated recombination and is restricted to the RPE. The scale bars in **G** and **H** indicate 500 μm while those in the insets indicate 100 μm. GCL, ganglion cell layer; INL, inner nuclear layer; IPL, inner plexiform layer; IS, inner segment; ONL, outer nuclear layer; OPL, outer plexiform layer; OS, outer segment; RPE, retinal pigment epithelium; Tam, tamoxifen.

As shown in Figure 2, we observed robust activation of mG fluorescence signal specifically in the RPE following the IP tamoxifen induction regimen. RPE flatmounts showed that more than 99% of RPE cells underwent recombination in mice started on tamoxifen either on PD21 (99.7%, *n* = 4) or PD50 (99.3%, *n* = 7), with the few un-recombined cells existing mainly in the peripheral retina (Figure 2, A–D). By contrast, conversion of the fluorescence signal was virtually absent in vehicle-treated animals (Figure 2, E and F). The level of tamoxifen-independent activation was 0.28% at PD64 (range 0.12%–0.55%, *n* = 6). Tamoxifen-dependent conversion was specific to the RPE as shown in retina cryosections, where green fluorescence was uniformly present in the apical and basal membranes of the RPE but absent throughout the neural retina (Figure 2, G and H, and Supplemental Figure 2). The results obtained with the chow delivery method closely mirrored those of the IP tamoxifen experiment with nearly complete red to green fluorescence conversion of the RPE in animals treated with tamoxifen starting on PD21 (99.8%, *n* = 2) or on PD50 (99.9%, *n* = 4) (Supplemental Figure 3, A–D). Again, we observed conversion only within the RPE (Supplemental Figure 3, E and F). Despite robust tamoxifen-dependent reporter conversion, we were unable to detect CreER^T2^ protein by Western blotting of whole eye lysates or isolated RPE from *Cre^+/–^* or *Cre^+/+^* mice using 2 commercially available antibodies. This apparently low Cre expression could have contributed to the minimal tamoxifen-independent activity we observed.

The RPE-restricted Cre expression in the *Rpe65^CreERT2^* mice is consistent with single-cell RNA-Seq data showing that the *RPE65* promoter is active only in the RPE (32, 44, 45). However, some previous immunohistochemical studies have suggested that RPE65 is also expressed in cone photoreceptors (46–48, but see ref. 49), which raised the possibility that Cre recombinase activity could be found in cones. To thoroughly examine this possibility, we prepared retina cryosections (Supplemental Figure 4A) as well as whole retina flatmounts (Supplemental Figure 4, B and C) from mice treated with the IP tamoxifen regimen starting on PD21 and stained them with peanut agglutinin to demarcate cone photoreceptors. We did not observe green fluorescence associated with cone photoreceptors or in any other regions of the neural retina, which confirmed the RPE specificity of Cre recombinase expression within the retina.

Because some RPE-enriched proteins, such as BEST1 (50) and MCT3 (51), are also expressed in the brain (particularly in the choroid plexus epithelium), we investigated Cre activity in brain sections from *Cre^+/–^*, mT/mG heterozygous mice treated with the IP tamoxifen regimen starting on PD21. No fluorescence conversion was observed in any region of the brain examined, including the choroid plexus epithelium (Supplemental Figure 5, A and B), thus confirming the lack of off-target activity, as predicted by our analysis of murine single-cell RNA-Seq data.

We compared the Cre-mediated recombination performance of the *Rpe65^CreERT2^* mouse to a commonly used inducible RPE Cre mouse line, the tet-ON PVMD2-rtTA::tetO-PhCMVcre (*VMD2-Cre*) mouse (27), using the mT/mG reporter line. Mice (heterozygous for both *VMD2-Cre* and the mT/mG reporter alleles) were gavaged with doxycycline or vehicle (water) on PD21 for 2 consecutive days (27) and then maintained under normal vivarium conditions for 2 weeks to allow time for degradation of the mT produced prior to induction. Cre recombination efficiency was then assessed by fluorescence microscopy of RPE flatmounts. We observed mosaic Cre activity in these mice as evidenced by the patchy conversion of fluorescence signal, particularly in the peripheral RPE (Supplemental Figure 6, A–D). Moreover, we found that a majority of vehicle-treated animals exhibited RPE cell fluorescence conversion comparable in extent to doxycycline-treated animals (Supplemental Figure 6, E–H). This mosaicism and doxycycline-independent Cre activity are consistent with prior reports (52, 53) and are in marked contrast to the virtually complete and highly tamoxifen-dependent Cre activity observed for the *Rpe65^CreERT2^* mouse.

### Impact of the KI locus on visual cycle function.

**Figure 3:**
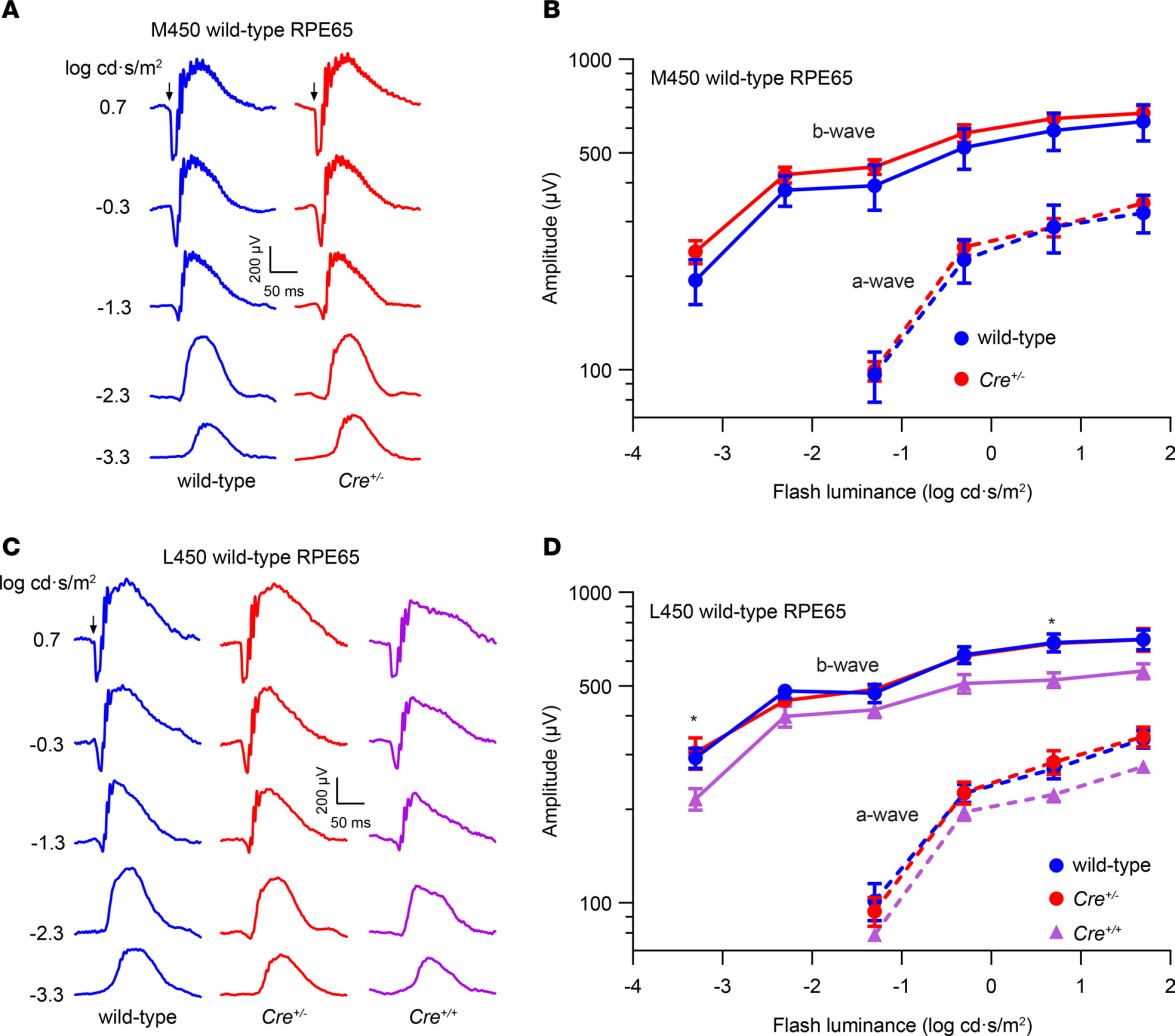
Impact of the KI allele on retinal function in Rpe65^CreERT2^ mice. (**A**) Representative scotopic ERG waveforms from a PD30 wild-type mouse and a *Cre^+/–^* littermate harboring the M450 variant of wild-type RPE65. (**B**) Summary luminance-response functions for the dark-adapted ERG a-waves and b-waves in wild-type (M450 RPE65, *n* = 5) and *Cre^+/–^* (*n* = 9) littermates. Data points indicate the mean ± SEM. Two-way repeated measures ANOVA did not reveal a significant effect of genotype on either a-wave (*F*_1,12_ = 0.14, *P* = 0.72) or b-wave (*F*_1,12_ = 0.74, *P* = 0.41) response. (**C**) Representative scotopic ERG waveforms from a representative PD30 wild-type mouse and a *Cre^+/–^* littermate harboring L450 wild-type RPE65 as well as a *Cre^+/+^* mouse with the same genetic background. (**D**) Summary luminance-response functions for the dark-adapted ERG a-waves and b-waves in wild-type (L450 RPE65, *n* = 6), *Cre^+/–^* (*n* = 4), and *Cre^+/+^* (*n* = 8) littermates. Data points indicate the mean ± SEM. Two-way repeated measures ANOVA revealed a significant effect of genotype on b-wave response (*F*_2,15_ = 4.62, *P* = 0.03) and a borderline significant effect on a-wave response (*F*_2,15_ = 3.30, *P* = 0.07). Multiple comparisons tests using Tukey’s correction showed significant (*P < 0.05) differences between wild-type and *Cre^+/+^* mice but no significant differences between wild-type and *Cre^+/–^* mice.

As described above, we observed that RPE65 expression from the KI allele was markedly lower than that from the native *Rpe65* gene. Because RPE65 is the nonredundant retinoid isomerase of the classical visual cycle, the diminished RPE65 levels might impair visual chromophore synthesis in *Rpe65^CreERT2^* mice. To test this hypothesis, we recorded scotopic electroretinograms (ERGs) from wild-type and *Rpe65^CreERT2^* mice following an overnight dark adaptation, as rod-mediated photoresponses are a sensitive indicator of visual chromophore deficiency (54). Despite the reduced level of RPE65 in *Cre^+/–^* mice, we observed that their rod-driven ERG responses were indistinguishable from wild-type control animals regardless of whether the animals expressed the less active (M450) or more active (L450) variants of wild-type RPE65 (Figure 3). Interestingly, dark-adapted ERGs from *Cre^+/+^* mice were relatively normal, although significantly reduced compared with wild-type mice (Figure 3, C and D), confirming that active RPE65 is produced from the *Rpe65^CreERT2^* allele.

**Figure 4:**
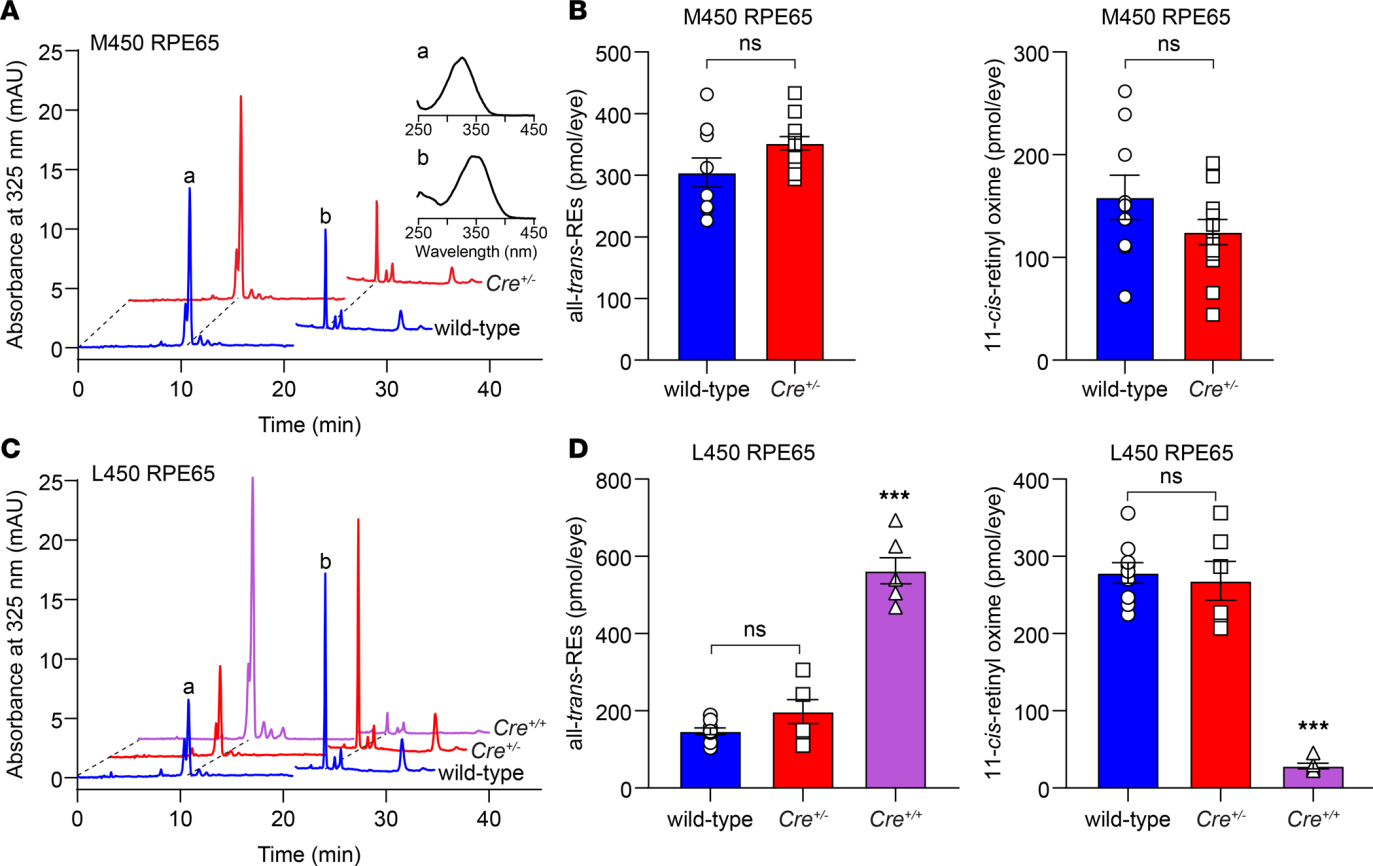
In vivo visual chromophore recovery in Rpe65^CreERT2^ mice following a photobleach. (**A**) Representative HPLC chromatograms showing separation of retinoids extracted from the eyes of a PD30 wild-type mouse (M450 wild-type *Rpe65*) and a *Cre^+/–^* littermate. The extracts were obtained after an in vivo 5000 lux × 10 minutes light exposure that bleached approximately 95% of rhodopsin followed by 4 hours of dark adaptation. Peaks “a” and “b” were identified as all-*trans*-retinyl esters and 11-*cis*-retinal oxime based on their retention times and absorbance spectra (inset). A solvent change artifact at approximately 21 minutes was omitted from all the chromatograms for clarity. (**B**) Quantification of all-*trans*-retinyl esters (left) and 11-*cis*-retinal oxime (right) in the extracts did not reveal a significant difference between the 2 groups (304.3 ± 23.6 vs. 352 ± 10.9, *P* = 0.09; and 158.5 ± 21.5 vs. 124.6 ± 12.2, *P* = 0.19, respectively). Statistical significance was assessed with 2-tailed *t* tests. (**C**) Representative HPLC traces showing separation of retinoids extracted from the eyes of a PD30 wild-type mouse (L450 wild-type *Rpe65*) and a *Cre^+/–^* littermate as well as an age-matched *Cre^+/+^* mouse with the same genetic background. (**D**) Quantification of all-*trans*-retinyl esters (left) and 11-*cis*-retinal oxime (right) in the extracts did not reveal a significant difference between wild-type (L450, *n* = 9) and *Cre^+/–^* (*n* = 6) mice (147.2 ± 8.8 vs. 197.9 ± 31.1, *P* = 0.29; and 278.5 ± 13.3 vs. 268.3 ± 25.2, *P* = 0.89, respectively). All-*trans*-retinyl esters (562.6 ± 33.8) and 11-*cis*-retinal oxime (28.5 ± 3.6) levels in *Cre^+/+^* mice were significantly different from both wild-type and *Cre^+/–^* mice (***P < 0.0001). Each point represents data from a single mouse. Bars indicate means ± SEM. Statistical significance was assessed by 1-way ANOVA followed by Tukey’s multiple comparisons test. ns, not statistically significant (*P* > 0.05).

To study the impact of the KI allele on visual cycle kinetics, we exposed wild-type and *Cre^+/–^* mice to intense light (5000 lux for 10 minutes) sufficient to bleach more than 95% of rhodopsin, then monitored rhodopsin recovery through HPLC analysis of 11-*cis*-retinal levels after a 4-hour dark adaptation period. We observed that 11-*cis*-retinal regeneration appeared slower in *Cre^+/–^* mice (M450 RPE65) as compared with wild-type (M450 RPE65) controls (Figure 4, A and B), although this difference did not reach statistical significance. This minor slowing is consistent with reports for mice with the M450 variant of RPE65, showing that visual cycle activity is rate limited by the expression level of RPE65 (55, 56). Accordingly, we hypothesized that *Cre^+/–^* mice with an L450 copy of *Rpe65* would display enhanced visual cycle kinetics. Indeed, *Cre^+/–^* (L450) mice exhibited greater than 2-fold higher 11-*cis*-retinal regeneration compared with *Cre^+/–^* (M450) mice (Figure 4, B–D). Moreover, there was only a 4% (nonsignificant) difference in 11-*cis*-retinal regeneration between wild-type (L450 RPE65) and *Cre^+/–^* (L450 RPE65) mice. In both models, retinyl esters (the substrates of RPE65) appeared somewhat increased in *Cre^+/–^* mice compared with their wild-type counterparts, although the differences were not statistically significant (Figure 4, A–D). By contrast, 11-*cis*-retinal recovery was significantly reduced, and all-*trans*-retinyl esters were significantly elevated in *Cre^+/+^* mice, consistent with the much lower expression of RPE65 in these mice (Figure 4, C and D). Collectively, these results show that visual cycle activity is not significantly affected in heterozygous *Rpe65^CreERT2^* mice despite weak expression of RPE65 protein from the KI allele (Figure 1C).

### Impact of Cre induction on retinal and RPE structure and function in Rpe65^CreERT2^ mice.

Previous studies have shown that the RPE is susceptible to Cre-dependent toxicity (29, 30). This toxicity is attributable either to the action of Cre on *LoxP*-like sites in the genome, resulting in progressive genetic lesions and cellular dysfunction, or to gene dysfunction resulting from insertion of the Cre-encoding sequence. To test for potential Cre-related toxicity in the *Rpe65^CreERT2^* mice, we treated *Cre^+/–^* mice or wild-type littermate controls with the 5-day IP tamoxifen regimen starting on PD21 and then assessed retinal structure and function at 2 or 4 months of age.

**Figure 5:**
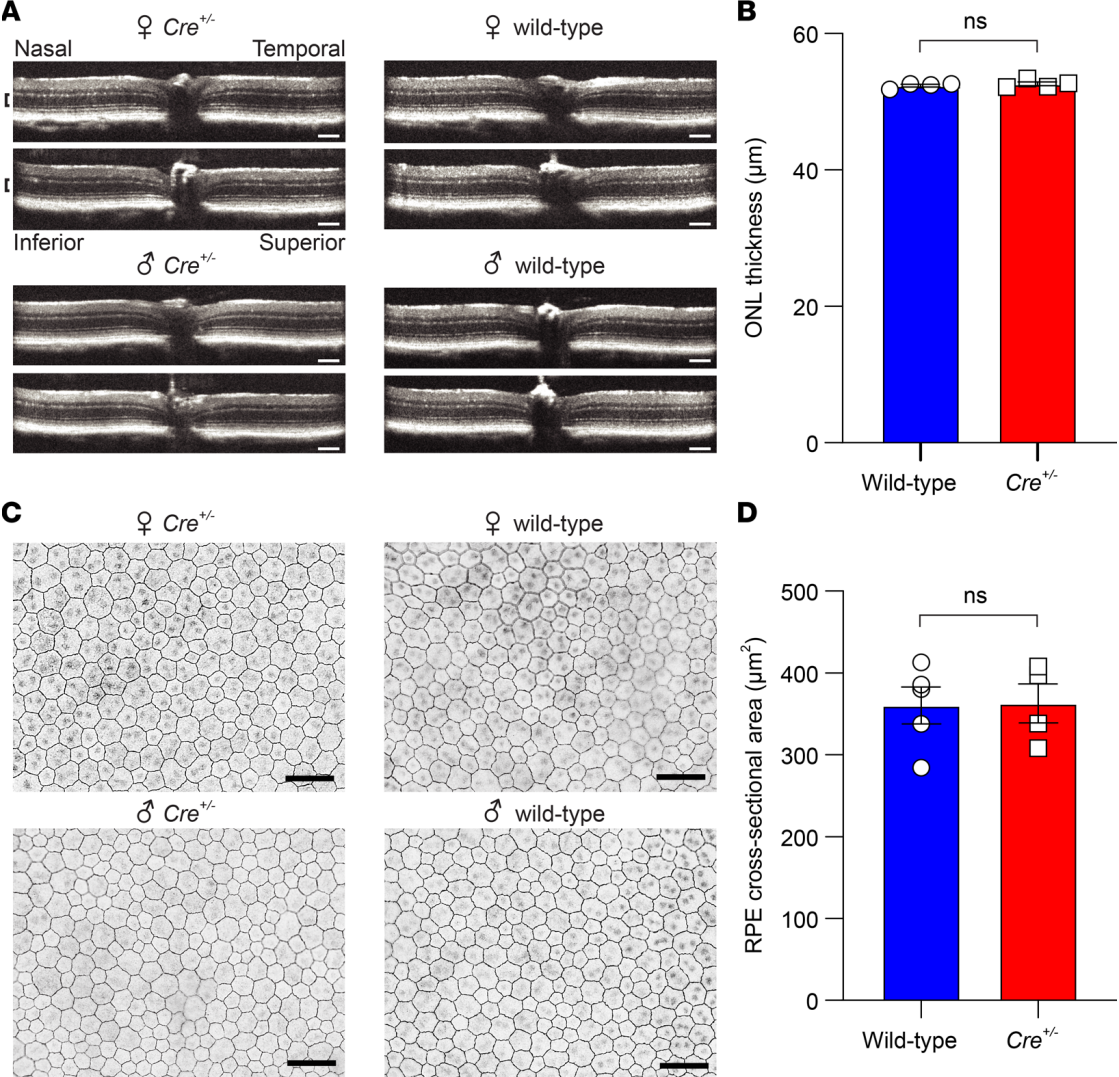
Impact of Cre induction on retinal and RPE structure in Rpe65^CreERT2^ mice. (**A**) Retinal OCT images from representative 4-month-old mice (M450 wild-type *Rpe65*) that had been treated with IP tamoxifen starting on PD21. The anatomical labeling in the upper left applies to all panels, and the scale bar indicates 100 μm. The laminar retinal structure is indistinguishable between wild-type and *Cre^+/–^* mice. (**B**) Quantification of the ONL thickness (demarcated by black brackets in **A**) showed no significant difference between wild-type (*n* = 4) and *Cre^+/–^* (*n* = 4) mice (52.4 ± 0.7 μm vs. 52.6 ± 0.7; *P* > 0.99, 2-tailed *t* test). (**C**) RPE flatmount images from the same mice as in **A** stained with an anti–ZO-1 antibody to allow visualization of the plasma membrane. The images were acquired within 1000 μm of the optic nerve. The scale bar indicates 50 μm. (**D**) Quantification of the RPE cross-sectional area showed no significant difference between wild-type (777 measured cells, *n* = 4) and *Cre^+/–^* (742 measured cells, *n* = 4) mice (360.1 ± 22.5 vs. 362.8 ± 23.8 μm^2^, respectively; *P* = 0.94, 2-tailed *t* test). Each point represents data from a single mouse. Bars indicate means ± SEM.

Examination of retinal histology at 2 months of age revealed comparable thickness of the outer nuclear layer (ONL), which contains the photoreceptor cell bodies, and the outer segment layer in wild-type and *Cre^+/–^* littermates, demonstrating that retinal structure was unaffected by the KI allele (Supplemental Figure 7). This conclusion was further supported by analysis of retinal structure through spectral domain optical coherence tomography (SD-OCT), which demonstrated a normal laminar appearance in 4-month-old *Cre^+/–^* mice that was indistinguishable from wild-type littermate controls (Figure 5A). The thickness of the ONL in *Cre^+/–^* mice remained indistinguishable from wild-type mice (Figure 5B). These results demonstrate that retinal structure was not affected by the presence of the KI allele or Cre recombinase activity. RPE structure was further evaluated through zonula occludens-1–immunostained (ZO-1–immunostained) RPE flatmounts, which allowed visualization of the cell membranes. Prior studies have shown that prolonged Cre activity can result in alterations in RPE morphology, including changes in cell size and a loss of the typical polygonal appearance (29, 30). In contrast to these findings, we observed that the RPE cells of 4-month-old *Cre^+/–^* mice exhibited normal size and morphology indistinguishable from the RPE of wild-type (M450 RPE65) littermates (Figure 5, C and D).

**Figure 6:**
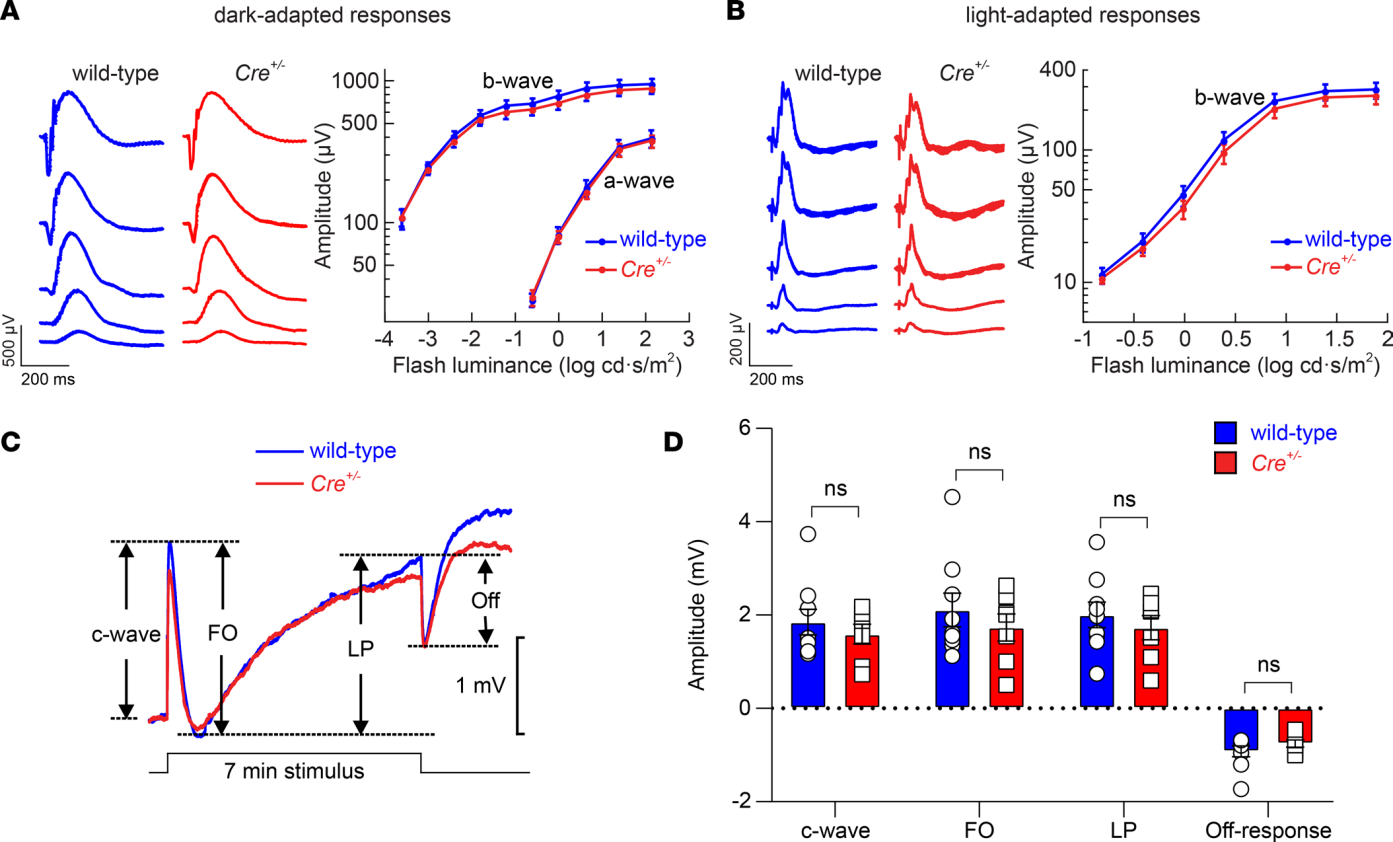
Impact of Cre induction on retinal and RPE function in Rpe65^CreERT2^ mice. (**A**) Representative dark-adapted ERG waveforms (left) and average (± SEM) a-wave and b-wave amplitudes (right) for 4-month old *Cre^+/–^* mice (*n* = 7) or wild-type (M450 wild-type *Rpe65*) littermates (*n* = 9) that were administered the 5-day IP tamoxifen induction regimen starting on PD21. Two-way repeated measures ANOVA did not reveal a significant effect of genotype on either a-wave (*F*_1,14_ = 0.04, *P* = 0.84) or b-wave (*F*_1,14_ = 0.38, *P* = 0.55) amplitudes. (**B**) Representative light-adapted flash ERG waveforms (left) and average (± SEM) b-wave amplitudes (right) for 4-month old *Cre^+/–^* mice or wild-type littermates (same animals as in **A** that were administered the 5-day IP tamoxifen induction regimen on PD21). Two-way repeated measures ANOVA did not reveal a significant effect of genotype on b-wave responses (*F*_1,14_ = 0.40, *P* = 0.54). (**C**) Representative dc-ERG waveforms obtained from a *Cre^+/–^* mouse or a wild-type littermate in response to a 7-minute light stimulus. The main dc-ERG components are labeled on the graph. FO, fast oscillation; LP, light peak. (**D**) The amplitudes of the major dc-ERG components were not significantly different between wild-type mice (*n* = 9) and *Cre^+/–^* littermates (*n* = 7) as assessed by multivariate ANOVA: c-wave (*F*_1,14_ = 0.50, *P* = 0.49), FO (*F*_1,14_ = 0.61, *P* = 0.45), LP (*F*_1,14_ = 0.51, *P* = 0.49), and off-response (*F*_1,14_ = 1.25, *P* = 0.28). The same groups of mice were used for both standard and dc-ERG analyses. Each point represents data from a single mouse. Bars indicate mean ± SEM.

Next, we evaluated potential pathological effects of Cre induction on retinal and RPE function. We used strobe flash ERGs to examine the response properties of the outer retina at 4 months of age in wild-type (M450 RPE65) and *Cre^+/–^* littermates that both received the IP tamoxifen regimen on PD21. Figure 6, A and B, show representative ERGs obtained under dark-adapted and light-adapted conditions from wild-type and *Cre^+/–^* littermates approximately 3 months after they had been given tamoxifen. We observed that the waveforms for the 2 groups were comparable. When the major components were quantified, there was no significant difference between wild-type and *Cre^+/–^* mice in terms of dark-adapted ERG a-wave or b-wave amplitudes or light-adapted cone ERG b-wave amplitudes. Next, we examined direct current ERGs (dc-ERGs) recorded in response to a light stimulus of 7-minute duration to assess the impact of the KI sequence and/or Cre activity on the electrical properties of the RPE (57). Figure 6C shows that the average dc-ERG responses of wild-type and *Cre^+/–^* mice overlapped. When the major dc-ERG components were quantified for wild-type and *Cre^+/+^* mice in the individual waveforms, there was no significant difference for any of them (Figure 6D). Preservation of the c-wave and other dc-ERG components indicates that the major ion currents of the RPE as well as its tight junction structure are unperturbed in *Rpe65^CreERT2^* mice (58, 59).

Taken together, these results demonstrate that *Rpe65^CreERT2^* mice do not exhibit signs of Cre-related toxicity when treated with a standard tamoxifen induction regimen that produced high-level *LoxP* recombination.

## Discussion

In this work, we describe a new approach for generation of an RPE-specific Cre mouse line that relies on cotranslational expression of CreER^T2^ from the native *Rpe65* gene. In accordance with the known expression profile of *Rpe65* (60–62), we observed nearly complete Cre-mediated recombination throughout the RPE cell layer and a lack of Cre recombinase activity elsewhere in the retina or in the brain. Induction of Cre recombinase was highly tamoxifen dependent and could be achieved with both IP injection and chow diet modes of tamoxifen administration. Additionally, we did not detect any pathological effects in the retina or RPE resulting from Cre expression in this mouse line up to 4 months of age. Thus, development of the *Rpe65^CreERT2^* mouse line has overcome the key limitations of previously reported RPE-targeted Cre mice; specifically, mosaic Cre expression, inducer-independent Cre activation, off-target Cre expression, and Cre-mediated RPE cell toxicity. It is noteworthy that protein expression from the *Rpe65^CreERT2^* allele (both RPE65 and CreER^T2^) was lower than we anticipated, which could have contributed to the low basal Cre activity level.

Although consistent with retinal single-cell RNA-Seq data (32, 44, 45), our demonstration that *Rpe65* promoter-driven Cre recombinase activity is absent in cone photoreceptors is notable in light of immunohistochemical data describing RPE65 expression within cones (46–48). However, such cone expression was not detected in another immunohistochemical study utilizing monoclonal RPE65 antibodies (49), and a recent report did not find a functional role for RPE65 in cone photoreceptors (63). The *Rpe65^CreERT2^* mouse provides a sensitive test for *Rpe65* promoter activity in cones because Cre expression driven by this promoter for even a limited time during the induction period would lead to irreversible fluorescent marking of the cone cell. The absence of Cre recombinase activity in cones that we have observed in the present work therefore suggests that RPE65 is not expressed in mouse cones under the conditions we have studied.

Mosaic Cre expression has been a particularly problematic issue associated with the existing RPE-targeted Cre mice reported to date. With one exception, these reported Cre lines were generated using random-integration transgenic approaches, as opposed to the targeted KI strategy used in this work, and are therefore susceptible to transgene epigenetic silencing and consequent mosaic Cre expression (38). Recently, a *BEST1*-CreER^T2^ mouse model was reported in which a single copy of the transgene was targeted to the *Rosa26* locus in an attempt to avoid this silencing issue (31). Despite an improvement in recombination efficiency, Cre activity remained somewhat mosaic in these mice, suggesting that this property could be intrinsic to the *BEST1* promoter. Additionally, these mice exhibited Cre activity within a fraction of Müller glia. By contrast, the *Rpe65* KI approach used in the current work avoided mosaicism while achieving strict RPE specificity.

Another shortcoming is inducer-independent Cre recombinase activity as we and others (27) have observed for the tet-ON *VMD2-Cre* mouse. Although such constitutive expression has been leveraged in recent studies (64), it is generally undesirable, and the approach we have used here provides a definite improvement in controlling Cre recombinase activity, allowing reliable activation in adult mice. We note that the mT/mG reporter allele has a higher threshold for basal recombination as compared with some other reporter lines (42). Depending on the properties of the floxed allele of interest, the level of recombination (ligand dependent or independent) achieved with the *Rpe65^CreERT2^* mouse could be higher or lower than what we have observed with the mT/mG reporter.

The low expression of RPE65 from the *Rpe65^CreERT2^* allele raised a concern that the visual cycle might be impaired in these mice. Indeed, *Cre^+/+^* mice exhibited reductions in dark-adapted ERG responses that were attributable to substantially slowed visual cycle kinetics. *Therefore, it is not recommended that homozygous Rpe65^CreERT2^ mice be used for typical LoxP recombination studies*. However, *Cre^+/–^* mice had normal ERG and visual chromophore recovery profiles, indicating that they are suitable for studies that require the visual cycle to be fully intact. We also show that further enhancement of visual cycle activity, for example as needed for light damage studies, can be achieved by introduction of a copy of L450 wild-type *Rpe65* as described previously (55, 56, 65). For a majority of studies, we expect that heterozygous *Rpe65^CreERT2^* mice with an M450 copy of wild-type *Rpe65* will be suitable. Our quantitative PCR data indicated that impaired transcription from the KI allele is not likely to be the major cause of the reduced RPE65 expression level. Inefficient ribosome skipping at the P2A sequence is also an unlikely contributor as we did not detect higher molecular weight fusion protein species by Western blotting. The non-native C-terminal residues present on RPE65 expressed from the KI allele could target it for degradation. Indeed, the chain length of RPE65 is highly conserved (60), and an additional non-native C-terminal sequence may impair protein stability and contribute to the diminished protein level.

*Rpe65* is a developmentally regulated gene with the transcript and protein first becoming detectable in rats at embryonic day 18 and PD4, respectively (66, 67). Although we did not investigate Cre activation at points earlier than PD21, it is possible that induction of Cre activity would be most effective starting on PD4 onward, since this is the starting point for RPE65 protein synthesis. Potential use of this mouse line at time points before PD21 will require additional validation studies.

In summary, we expect this new *Rpe65^CreERT2^* mouse line will be a valuable tool for the study of retinal/RPE function. Many avenues of investigation are expected to benefit, including the ability to distinguish the roles of abundant mRNAs common to the RPE and photoreceptors, to discern the relative importance of classical versus photic retinoid cycles in visual function, and to generate murine models to study the pathophysiology and treatment of retinal degenerative disorders, such as age-related macular degeneration.

## Methods

### Animal husbandry.

Animals were housed in a standard 12-hour light/12-hour dark cycle environment, fed standard soy protein–free rodent chow diet (Envigo Teklad 2020X, with the exception of experiments involving tamoxifen induction), provided water ad libitum, and housed in plastic cages with standard corncob rodent bedding and 6-gram nestlets (Ancare).

### Generation of the Rpe65^CreERT2^ mouse.

Generation of the *Rpe65^CreERT2^* mouse was performed by Ingenious Targeting Laboratory using standard techniques, under a fee-for-service contract. Briefly, FLP C57BL/6 embryonic stem cells were electroporated with a targeting construct encoding a P2A-CreER^T2^ sequence fused in-frame with exon 14 of the *Rpe65* gene as well as an FRT-flanked neomycin cassette placed immediately after the noncoding exon 15. Properly targeted stem cells were identified by neomycin selection and then expanded and screened by PCR analysis. The neomycin cassette was deleted during colony expansion. Properly targeted stem cells were microinjected into BALB/c blastocysts. The resulting chimeras with a high-percentage black coat color were mated to C57BL/6N wild-type mice to generate germline Neo cassette–deleted mice, which were further mated with wild-type C57BL/6N mice to eliminate the FLP transgene.

### Animal breeding and locus sequencing.

The *Rpe65^CreERT2^* mice obtained from Ingenious Targeting Laboratory were backcrossed with wild-type C57BL/6J mice for 2 generations to eliminate the rd8 mutation found in the F2 progeny. The integrity of the entire targeted region including the 5′ and 3′ homology arms was verified by PCR-amplifying the region of interest in 7 overlapping fragments that were each Sanger-sequenced using appropriate primers. The genome sequence exactly matched that of the targeting construct. *Rpe65^CreERT2^* mice on a C57BL/6J background have been deposited with The Jackson Laboratory (JAX 035973).

To assess Cre recombinase activity, *Rpe65^CreERT2^* mice were crossed with homozygous B6.129(Cg)-Gt(ROSA)26Sor*^tm4(ACTB–tdTomato,–EGFP)Luo^*/J mice (mT/mG, JAX 007676) to generate mice heterozygous for the *Rpe65^CreERT2^* and mT/mG alleles. The *Rpe65^CreERT2^* mice were also crossed with 129S1 (JAX 002448) mice for 2 generations to introduce a copy of the L450 variant of RPE65, which allowed assessment of visual cycle activity in the setting of a fully active copy of RPE65. In some cases, *Rpe65^CreERT2^* mice on a mixed C57BL/6J and 129S1 background were used for mT/mG reporter studies, although the genetic background did not affect the experimental outcomes. *VMD2-Cre* (PVMD2-rtTA::tetO-PhCMVcre) mice (27) were crossed with mT/mG mice and the offspring used to assess Cre recombinase activity.

### Genotyping.

Ear punch samples were digested at 55°C overnight in 150 μL of DirectPCR (tail) buffer (Viagen Biotech) containing proteinase K (Viagen Biotech) at a concentration of 4 μg/mL. The peptidase was inactivated by incubating the digested samples at 85°C for 1 hour, and the supernatant containing genomic DNA was collected. Genotyping was performed by standard PCR using GoTaq Green Master Mix (Promega) and a Bio-Rad T100 thermal cycler. Primers and cycling parameters used for each region of interest are shown in Supplemental Table 2. To distinguish between the M450 and L450 variants of *Rpe65*, a 17.5 μL aliquot of the PCR product generated using primers Rpe65f and Rpe65r (Supplemental Table 2) was mixed with 2 μL of CutSmart buffer and 0.5 μL of MwoI restriction enzyme (New England Biolabs) and incubated at 37°C for 1 hour. Reactions were analyzed by agarose gel electrophoresis.

### Real-time PCR for mRNA quantification.

Total RNA from the RPE layer of wild-type, *Cre^+/–^*, and *Cre^+/+^* mice was extracted using a previously published protocol (68). After removing the anterior segment and retina, the resulting posterior eye cup was transferred into a tube containing 200 μL of RNAprotect Cell Reagent (QIAGEN, 76526). The eye cup was incubated for 10 minutes at room temperature with gentle agitation performed every 2 minutes. After removing the eye cup from the tube, the RPE was pelleted by centrifugation for 5 minutes at 600*g* and subjected to total RNA extraction using an RNeasy Mini Kit (QIAGEN). Then, cDNA was synthesized with a high-capacity RNA-to-cDNA kit (Applied Biosystems, Thermo Fisher Scientific). Real-time PCR was performed using iQ SYBR Green Supermix (Bio-Rad) with the following *Rpe65* primers: forward, 5′-AAGGCTCCTCAGCCTGAAGTCA-3′; reverse, 5′-GAGAACCTCAGGTTCCAGCCAT-3′. *Gapdh* was used as a housekeeping gene for normalization. Triplicate real-time PCR reactions were performed for each animal.

### Cre induction experiments.

For experiments involving *Rpe65^CreERT2^* mice, Cre recombinase activity was induced by IP injection of tamoxifen (MilliporeSigma, T5648) dissolved in corn oil vehicle (20 mg/mL stock) for 5 consecutive days (1 mg on days 1 through 3 and 2 mg on days 4 and 5) or by feeding mice tamoxifen-loaded chow diet (250 mg tamoxifen/kg chow, Envigo, TD.130856) for 3 weeks. Tamoxifen treatment was started at either PD21 or PD50. For experiments involving tet-ON *VMD2-Cre* mice, Cre recombinase activity was induced by administering 8 mg of doxycycline hyclate (MilliporeSigma, D9891) in 100 μL of water by oral gavage for 2 consecutive days starting on PD21.

### Western blotting.

Following euthanasia, enucleated eyes were dissected and processed using a previously published protocol (69). After removing the anterior segment, the retina and RPE were isolated and extracted separately to obtain the corresponding tissue-specific protein lysates. Protein concentrations were measured with a Pierce Rapid Gold BCA Protein Assay Kit (Thermo Fisher Scientific) following the manufacturer’s instructions. The resulting protein samples were mixed with LDS sample buffer (Thermo Fisher Scientific) and NuPAGE reducing agent (Thermo Fisher Scientific). Then, the samples were separated using a NuPAGE 4%–12% Bis-Tris gel (Thermo Fisher Scientific) and transferred onto 0.45 μm nitrocellulose membranes (Thermo Fisher Scientific). The membranes were blocked with 5% (*w/v*) nonfat milk in phosphate-buffered saline (PBS) containing 0.1% (*v/v*) Tween 20 (PBS-T) for 30 minutes and then incubated with an anti-RPE65 monoclonal antibody (40) (1:1000), anti-Cre antibodies (1:1000; Novus, NB100-56134 or MilliporeSigma, MAB3120), or an anti–β-actin antibody (1:1000; Cell Signaling Technology, 4970S) overnight at 4°C. After overnight incubation, the membranes were washed 3 times with PBS-T for 5 minutes each and then incubated with either an anti-mouse IgG-HRP antibody (1:10,000; Cell Signaling Technology, 7076S) or an anti-rabbit IgG-HRP antibody (1:10,000; Cell Signaling Technology, 7074S) for 1 hour at room temperature. The protein bands were visualized using an Odyssey Fc imaging system (LI-COR) after exposure to SuperSignal West Pico Chemiluminescent Substrate (Thermo Fisher Scientific). ImageJ (NIH) was used for quantification of RPE65 expression levels.

### RPE flatmount preparation and ZO-1 immunostaining.

Following euthanasia, mouse eyes were fixed with 4% paraformaldehyde in PBS for 30 minutes at room temperature, then washed 3 times in PBS for 10 minutes each. To make RPE flatmounts, the anterior segment and retina were removed from the posterior eye cup under a dissecting microscope, and 4 radial cuts were made toward the optic nerve head to flatten the eye cup. For the evaluation of mT and mG expression in the RPE, RPE flatmounts were mounted with ProLong Gold Antifade mounting medium (Thermo Fisher Scientific) and imaged using Texas red and GFP filters and a 10× objective with a Keyence BZ-X810 All-in-One fluorescence microscope.

To evaluate ZO-1 immunostaining, RPE flatmounts were permeabilized in 0.5% Triton X-100 (MilliporeSigma) in PBS for 30 minutes, blocked in 3% BSA in PBS for 30 minutes, and then incubated with rabbit anti–ZO-1 polyclonal antibody (1:100; Invitrogen, Thermo Fisher Scientific, 61-7300) overnight at 4°C. The next day, samples were washed 3 times in PBS for 10 minutes each and then incubated with secondary antibody, Alexa Fluor 594–conjugated goat anti-rabbit IgG (1:200; Thermo Fisher, A-11012), for 2 hours at room temperature in the dark. Samples were washed 3 times in PBS for 10 minutes each, then mounted with ProLong Gold mounting medium and imaged as described above. These RPE flatmount images were acquired at 40× magnification with a Keyence BZ-X810 All-in-One fluorescence microscope.

### Retina flatmount preparation and cone photoreceptor staining.

Following euthanasia, mouse eyes were fixed with 4% paraformaldehyde in PBS (Santa Cruz Biotechnology) for 1 hour at room temperature and washed 3 times in PBS for 10 minutes each. To make retina flatmounts, the retina tissue was separated from the anterior segment and posterior eye cup under a dissecting microscope, and 4 radial cuts were made toward the optic nerve head to flatten the retina. To stain cone photoreceptors, retina flatmounts were permeabilized in 0.5% Triton X-100 (MilliporeSigma) in PBS for 30 minutes and incubated with Cy5-labeled peanut agglutinin (1:200; Vector Laboratories, CL-1075-1) in permeabilization solution containing 5% normal donkey serum (Abcam) overnight at 4°C. The next day, flatmounts were washed 3 times in PBS for 10 minutes each, then mounted with ProLong Gold mounting medium and imaged with Keyence BZ-X810 All-in-One fluorescence microscope.

### Retina cryosectioning and histochemistry.

Following euthanasia, mouse eyes were enucleated and dissected along the posterior margin of the limbus. The resulting eye cups were fixed for 20 minutes in PBS containing 4% (*w/v*) paraformaldehyde (MilliporeSigma) at room temperature. After fixation, the eye cups were incubated sequentially in PBS containing 10%, 20%, and 30% (*w/v*) sucrose (MilliporeSigma) for 30 minutes at room temperature. Then, the eye cups were infiltrated with a 2:1 mixture of PBS containing 30% sucrose and O.C.T. compound (VWR International) and frozen by immersion in dry ice. Retinal sections were cut at a thickness of 12 μm and stored at –80°C until use. The retinal sections were rehydrated with PBS and then mounted with VECTASHIELD Mounting Medium (Vector Laboratories) for direct imaging. To visualize cone photoreceptors, the retinal sections were permeabilized with 0.2% Triton X-100 (MilliporeSigma) in PBS for 30 minutes and then incubated with Cy5-labeled peanut agglutinin (1:200; Vector Laboratories, CL-1075-1) in permeabilization solution containing 5% normal donkey serum (Abcam) overnight at 4°C. The next day, the retinal sections were washed 3 times in PBS for 10 minutes each, then mounted with VECTASHIELD Mounting Medium for imaging. To visualize the RPE, the retinal sections were permeabilized with 0.2% Triton X-100 (MilliporeSigma) in PBS for 30 minutes, blocked with 5% goat serum (Thermo Fisher Scientific) in PBS, and then incubated with either an anti-RPE65 antibody (1:250; ref. 40) or an anti-ezrin antibody (1:400, Abcam, ab4069) in permeabilization solution containing 1% goat serum (Thermo Fisher Scientific) in PBS overnight at 4°C. The next day, the retinal sections were washed 3 times in PBS for 10 minutes each and then incubated with an Alexa Fluor 647–conjugated goat anti-mouse IgG (1:300; Thermo Fisher Scientific, A28181) for 2 hours at room temperature in the dark. The retinal sections were washed 3 times in PBS for 10 minutes each, then mounted with VECTASHIELD Mounting Medium for imaging. Fluorescence images were acquired with a Keyence BZ-X800 All-in-One fluorescence microscope.

### Brain cryosectioning.

Following euthanasia, the mouse brain was removed and immediately fixed in 4% paraformaldehyde in PBS for 48 hours. A total of 6 coronal slices were made with a scalpel from the frontal lobe to the cerebellum. Each of the slices was infiltrated via a graded series of increasingly concentrated sucrose solutions in PBS, i.e., 2 hours for each of the 10% (*w/v*) and 20% (*w/v*) sucrose solutions, then incubated overnight in 30% (*w/v*) sucrose at 4°C. Slices were then embedded in O.C.T. medium (Sakura Finetek) for cryosectioning. Sections (10 μm thickness) were obtained with a cryostat and stored at –80°C until needed for imaging studies. The images were acquired with a Keyence BZ-X800 All-in-One fluorescence microscope.

### Histology.

Following euthanasia, each mouse eye was cauterized to mark the superior pole, removed from the orbit with curved scissors, and immediately placed in Hartman’s fixative (MilliporeSigma) at room temperature. After 24 hours, the eyes were transferred to 70% *v/v* ethanol for shipping. Histology was performed by HistoWiz Inc. using a standard operating procedure and fully automated workflow. Samples were processed, embedded in paraffin, sectioned at 4 μm along the superior-inferior axis passing through the optic nerve, and stained with hematoxylin and eosin. After staining, sections were dehydrated and film-coverslipped using a Tissue-Tek-Prisma and Coverslipper (Sakura). Whole slide scanning (40×) was performed on an Aperio AT2 (Leica Biosystems).

### Image analysis.

Analysis of microscopy images was performed using ImageJ (70) or Aperio ImageScope (Leica Biosystems) software. RPE cells from ZO-1–immunostained retinal flatmounts were manually counted by an unbiased observer. The RPE cell density was estimated to be 7.3 cells/2500 μm^2^. The total surface area of the adult mouse retina was estimated to be approximately 17.8 mm^2^, which is consistent with prior literature values (71). Therefore, we calculated that there are approximately 52,000 RPE cells per mouse retina, consistent with prior estimates (72, 73). To avoid counting cells in the far periphery that had been damaged by removal of the anterior segment, only the inner 17 mm^2^ of the retina was used for analysis of recombination efficiency and tamoxifen-independent Cre activity. Therefore, a denominator of 49,663 RPE cells was used for all recombination calculations.

To estimate the number of RPE cells with tamoxifen-independent Cre activity, the red and blue channels were removed from the micrographs, and the green fluorescent cells were manually counted by an unbiased observer. To estimate the number of RPE cells that did not undergo fluorescence conversion, the red and green channels were adjusted to emphasize both signals. Then cells lacking green fluorescence signal, but still exhibiting red fluorescence, were manually counted. The few areas without red fluorescence signal represent cells that had dissociated during the RPE flatmount procedure; hence, they were not included in the cell count.

### OCT imaging.

Mouse retinas were analyzed in vivo with SD-OCT (Bioptigen). Briefly, the mice were first administered a mydriatic eye drop (1% tropicamide, Bausch & Lomb) and then anesthetized with an IP injection of a ketamine (100 mg/kg) and xylazine (10 mg/kg) mixture. The A-scan/B-scan ratio was set at 1200 lines. Five SD-OCT images scanned at 0 and 90 degrees were acquired in the B-mode, averaged, and saved as PDF files. ONL thickness was measured 500 μm from the optic nerve head in the superior, inferior, temporal, and nasal retina. Values from each eye quadrant were averaged to give an overall value per eye.

### Electroretinography.

Mice were studied using conventional flash ERG to evaluate outer retinal function with 2 protocols.

At the Cleveland Clinic Foundation, mice were anesthetized with an IP injection of a ketamine (80 mg/kg) and xylazine (16 mg/kg) mixture, and the pupils were dilated with eyedrops (1% tropicamide, 1% cyclopentolate, 2.5% phenylephrine HCl) after overnight dark adaptation (74). The cornea was anesthetized with an eyedrop of 1% proparacaine HCl, and the mouse was then placed on a temperature-controlled heating pad within a Faraday cage. Strobe flash stimuli were used to evoke responses generated by the outer retina following a published protocol (74). ERGs were recorded using a stainless steel wire electrode that contacted the corneal surface; needles placed in the cheek and tail served as reference and ground electrodes, respectively. Responses were differentially amplified (bandpass: 0.3 to 1500 Hz) and averaged with a UTAS system (LKC Technologies). Dark-adapted (scotopic) responses were obtained to a stimulus range of –3.6 to 2.1 log cd·s/m^2^. Light-adapted (photopic) responses were obtained to a stimulus range of –0.8 to 1.9 log cd·s/m^2^ that was superimposed on a steady achromatic background of 20 cd/m^2^. The a-wave amplitude was measured 8 ms after flash onset from the prestimulus baseline. The b-wave amplitude was measured from the a-wave trough to the peak of the b-wave or from the prestimulus baseline if the a-wave was not detectible.

At University of California, Irvine, ERGs were recorded with a Diagnosys Celeris rodent ERG device as previously described (75). Briefly, a mouse was anesthetized with an IP injection of a ketamine (80 mg/kg) and xylazine (10 mg/kg) mixture and placed on a heating pad at 37°C. Its corneas were moistened with 0.3% hypromellose eye lubricant (Genteal Tears Severe Dry Eye Lubricant Gel, Alcon). Light stimulation was produced by an in-house scripted stimulation series in Espion software (version 6; Diagnosys). Before scotopic recordings, the mice were dark-adapted overnight, and animal handling was performed under dim red light. After handling, the animals were kept in total darkness for 3 minutes. The eyes were stimulated with a green LED (peak 544 nm, bandwidth 160 nm) using a 6-step ascending flash intensity series ranging from –3.3 to 1.7 log cd·s/m^2^. The ERG signal was acquired at 2 kHz and filtered with a low-frequency cutoff at 0.25 Hz and a high-frequency cutoff at 300 Hz. Espion software automatically detected the ERG a-wave (first negative ERG component) and b-wave (first positive ERG component).

To assess RPE cell function, RPE-specific ERG components were examined using On-Off dc-ERG (57). dc-ERG responses were recorded from the corneal surface of the left eye using a capillary tube with Ag/AgCl filament (BF100-50-10; Sutter Instrument) and Hanks’ buffered salt solution (Cell Culture and Media Preparation Cores, Lerner Research Institute, Cleveland Clinic Foundation). An identical electrode was placed on the cornea of the unstimulated right eye as the reference electrode. The responses were differentially amplified (bandpass: 0 to 100 Hz) and sampled at 20 Hz, using LabScribe Data Recording Software (iWorx). Achromatic stimuli were delivered to the left eye from an optical channel using a Leica microscope illuminator and a 1-centimeter-diameter fiber-optic bundle with stimulus luminance of 2.4 log cd/m^2^. In each recording epoch, after a 30-second baseline, a shutter system (Uniblitz) was used to present a 7-minute-duration stimulus. Recording was continued after flash offset, so the total recording epoch was 10 minutes. The c-wave amplitude was measured from the prestimulus baseline to the c-wave peak. The FO amplitude was measured from the c-wave peak to the FO trough. The LP amplitude was measured from the FO trough to the asymptotic or the maximum value in the 7-minute stimulating-light-on period. The off-response amplitude was measured from the LP prior to stimulus offset to the trough of the off-response.

### Statistics.

Statistical analysis was carried out using GraphPad Prism version 9.0 or SPSS. Statistical methods are reported in the figure legends. A *P* value less than 0.05 was considered significant.

### Study approval.

Animal procedures were approved by the Institutional Animal Care and Use Committees at the VA Long Beach Healthcare System; the University of California, Irvine; or the Cleveland Clinic. All experimental protocols were conducted in accordance with the NIH *Guide for the Care and Use of Laboratory Animals* (National Academies Press, 2011), the recommendations of the American Veterinary Medical Association Panel on Euthanasia, and the Association for Research in Vision and Ophthalmology *Statement for the Use of Animals in Ophthalmic and Visual Research*.

## Author contributions

EHC, SS, HL, ZD, SB, MY, NSP, KP, and PDK designed experiments. EHC, SS, DEE, HL, ZD, MY, and PDK performed experiments. EHC, SS, HL, ZD, MY, NSP, and PDK analyzed data. SRR and SJF provided reagents. PDK supervised the project. EHC and PDK wrote the paper with feedback from all coauthors.

## Supplementary Material

Supplemental data

## Version 1 03/30/2021

In-Press Preview

## Version 2 05/10/2021

Electronic publication
