# Supplementary material for: An inducible Cre mouse for studying roles of the RPE in retinal physiology and disease
Source: JCI Insight. 2021 May 10;6(9):e146604. doi: 10.1172/jci.insight.146604 (PMC8262343; doi:10.1172/jci.insight.146604)
Supplement: Supplemental data [file jciinsight-6-146604-s028.pdf]

**SUPPLEMENTAL INFORMATION FOR**

# An inducible Cre mouse for studying roles of the RPE in retinal physiology and disease

Elliot H. Choi[1,2], Susie Suh[1,2], David E. Einstein[3,4], Henri Leinonen[1], Zhiqian Dong[1], Sriganesh Ramachandra Rao[5,6], Steven J. Fliesler[5,6], Seth Blackshaw[7], Minzhong Yu[8,9], Neal S. Peachey[8,9,10], Krzysztof Palczewski[1,3,11], and Philip D. Kiser[1,3,4,*]

[1] Gavin Herbert Eye Institute, Department of Ophthalmology, University of California, Irvine, CA 92697 USA

[2] Department of Pharmacology, Case Western Reserve University, Cleveland, OH 92697, USA

[3] Department of Physiology & Biophysics, University of California, Irvine, CA 92697, USA

[4] Research Service, VA Long Beach Healthcare System, Long Beach, CA 90822 USA

[5] Departments of Ophthalmology and Biochemistry and Neuroscience Graduate Program, University at Buffalo, The State University of New York, Buffalo, NY 14260 USA

[6] Research Service, VA Western New York Healthcare System, Buffalo, NY 14215 USA

[7] Department of Neuroscience, Johns Hopkins University School of Medicine, Baltimore, MD 21205, USA

[8] Department of Ophthalmic Research, Cole Eye Institute, Cleveland Clinic Foundation, Cleveland, OH 44195, USA

[9] Department of Ophthalmology, Cleveland Clinic Lerner College of Medicine of Case Western Reserve University, Cleveland, OH 44195, USA

[10] Research Service, Louis Stokes Cleveland VA Medical Center, Cleveland, OH 44106, USA

[11] Department of Chemistry, University of California, Irvine, CA 92697, USA

*Correspondence: Philip D. Kiser: Departments of Physiology & Biophysics and Ophthalmology, University of California, Irvine, CA and Research Service, VA Long Beach Healthcare System, Long Beach, CA; pkiser@uci.edu

**SUPPLEMENTAL FIGURES, FIGURE LEGENDS, AND TABLES**

# Figure S1

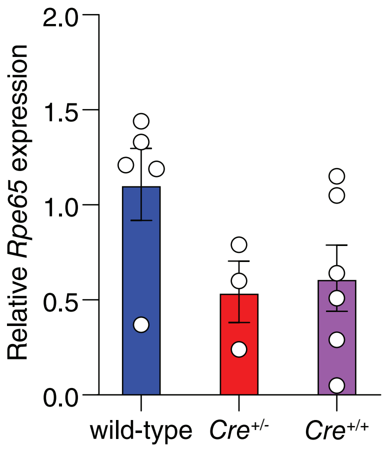

**Figure S1.** *Quantitative PCR analysis of Rpe65 mRNA expression. Rpe65* transcript levels were measured by RT-PCR and normalized to the *Gapdh* transcript levels. *Rpe65* was reduced by ~50% in *Cre*$^{+/-}$ (n = 3) and *Cre*$^{+/+}$ (n = 6) mice as compared to wild-type controls (n = 5), although these differences were not statistically significant as assessed by one-way ANOVA [$F_{(2,11)} = 2.671$, $P = 0.11$]. Each point represents data from a single mouse. Bars indicate mean ± SEM.

# Figure S2

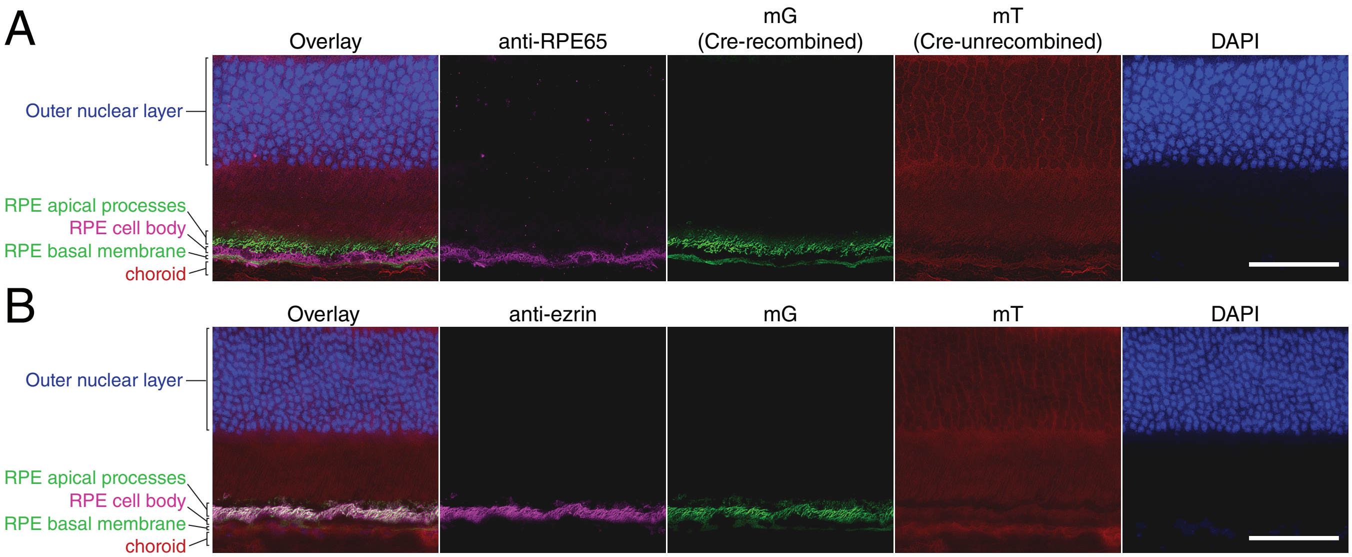
A
Overlay
anti-RPE65
mG
(Cre-recombined)
mT
(Cre-unrecombined)
DAPI
Outer nuclear layer
RPE apical processes
RPE cell body
RPE basal membrane
choroid
B
Overlay
anti-ezrin
mG
mT
DAPI
Outer nuclear layer
RPE apical processes
RPE cell body
RPE basal membrane
choroid

**Figure S2.** *Relationship of retinal mT and mG fluorescence to RPE cell markers.* **A**) A retina cryosection from a PD21 IP tamoxifen-treated *Cre*$^{+/-}$ *mT/mG*$^{+/-}$ mouse was co-stained with an anti-RPE65 Ab and DAPI. The leftmost image shows the overlaid fluorescence signals while the images to the right show the individual channels. The RPE65-associated fluorescence is localized to the cell body (1) in-between the mG signals associated with the basal and apical plasma membranes of the RPE. Note that the scattered specs in this image were also present in the secondary antibody-only control. The scale bar on the rightmost panel represents 50 $\mu$m. **B**) A retina cryosection from an PD21 IP tamoxifen-treated *Cre*$^{+/-}$ *mT/mG*$^{+/-}$ mouse was co-stained with an anti-ezrin antibody and DAPI. The leftmost image shows the overlaid fluorescence signals while the images to the right show the individual channels. The overlap between the mG and ezrin-associated fluorescence signals confirms the localization of mG to the RPE apical membrane (1). The scale bar on the rightmost panel represents 50 $\mu$m.

# Figure S3

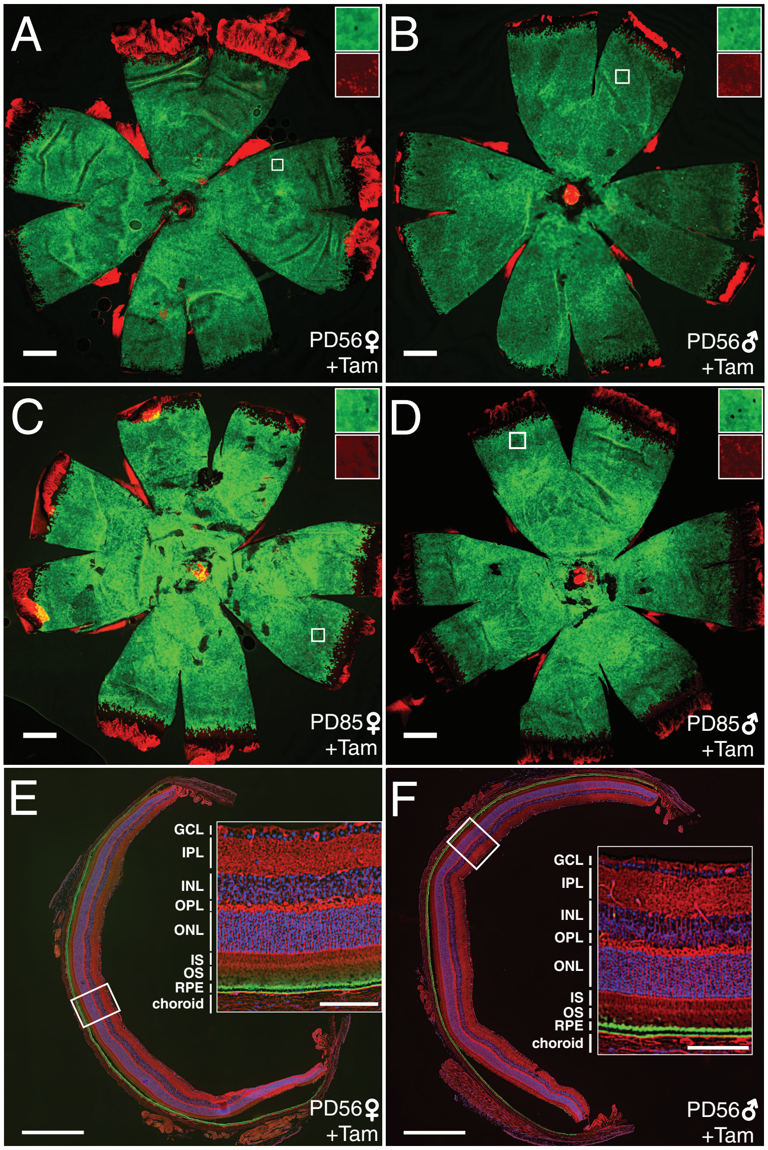
A
PD56♀
+Tam
B
PD56♂
+Tam
C
PD85♀
+Tam
D
PD85♂
+Tam
E
GCL
IPL
INL
OPL
ONL
IS
OS
RPE
choroid
PD56♀
+Tam
F
GCL
IPL
INL
OPL
ONL
IS
OS
RPE
choroid
PD56♂
+Tam

**Figure S3.** *Cre recombinase activity in Rpe65$^{CreERT2}$ mouse retina assessed with the mT/mG reporter.* Panels **A** and **B** show RPE flatmounts from *Cre*$^{+/-}$ female and male mice, respectively, that were fed tamoxifen chow diet for three weeks starting on PD21 (n = 2). The flatmounts were obtained two weeks after stopping the tamoxifen chow diet and imaged with a fluorescence microscope. Green fluorescence indicates cells where Cre-mediated recombination has occurred, whereas red fluorescence indicates un-recombined cells. Panels **C** and **D** show RPE flatmounts from representative *Cre*$^{+/-}$ female and male mice, respectively, that were fed tamoxifen chow diet for three weeks starting on PD50 (n = 4). The flatmounts were obtained two weeks after stopping tamoxifen chow diet. The insets in panels **A-D** show zoomed areas (marked by white squares) with green and red channels separately displayed to demonstrate the single cell resolution of the imaging method. Panels **E** and **F** show retina cryosections from *Cre*$^{+/-}$ female and male mice, respectively, that were fed tamoxifen chow for three weeks starting on PD21 (n = 2). The cryosections were obtained two weeks after stopping tamoxifen chow diet. The green label represents Cre-mediated recombination and is restricted to the RPE. Insets show zoomed views of the areas marked with rectangles with the retinal cell layers labeled. Abbreviations: GCL, ganglion cell layer; INL, inner nuclear layer; IPL, inner plexiform layer; IS, inner segment; ONL, outer nuclear layer; OPL, outer plexiform layer; OS, outer segment; RPE, retinal pigment epithelium. The scale bars in each panel indicate 500 μm while those in the insets in panels E and F indicate 100 μm. Tam – tamoxifen.

# Figure S4

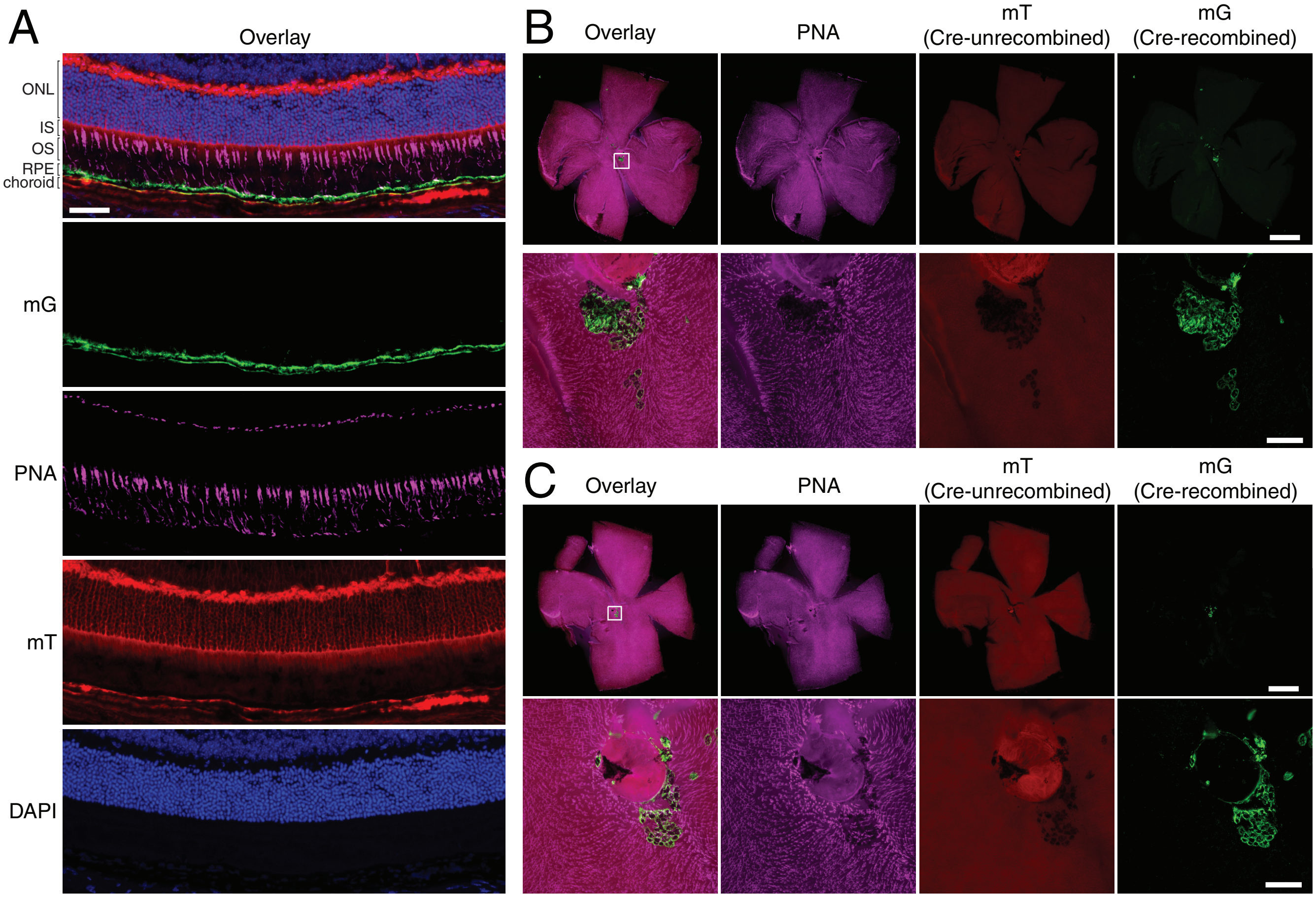
A
Overlay
ONL
IS
OS
RPE
choroid
mG
PNA
mT
DAPI
B
Overlay
PNA
mT
(Cre-unrecombined)
mG
(Cre-recombined)
C
Overlay
PNA
mT
(Cre-unrecombined)
mG
(Cre-recombined)

**Figure S4.** *Lack of mG fluorescence co-localization with cone photoreceptors.* **A**) Retina cryo-sections from Cre+/- mT/mG+/- mice treated with IP tamoxifen starting on PD21 were co-stained with peanut agglutinin, which selectively stains cone photoreceptors, and DAPI. The top image shows the composite fluorescence signal and those below in are the individual channels. No co-localization of the PNA and mG signals was observed. The scale bar represents 50 μm. Whole retina flatmounts from female (**B**) or male (**C**) $Cre^{+/-}$ $mT/mG^{+/-}$ mice treated with IP tamoxifen on PD21 were co-stained with PNA to allow visualization of cone photoreceptors. The retinae were imaged with their RPE-associated sides facing the camera. The leftmost images show the composite fluorescence signal while the images to the right show the individual channels. Zoomed images corresponding to the indicated box are shown below each whole-retina image. No mG signal is seen to co-localize with the PNA cone cell marker indicating that Cre recombinase in not expressed in cone photoreceptors in $Rpe65^{CreERT2}$ mice. The zoomed images show mG-positive RPE cells that remained adhered to the neural retina during the dissection procedure. The scale bars in the whole retina images represent 1000 μm while those in the bottom zoomed images represent 100 μm.

# Figure S5

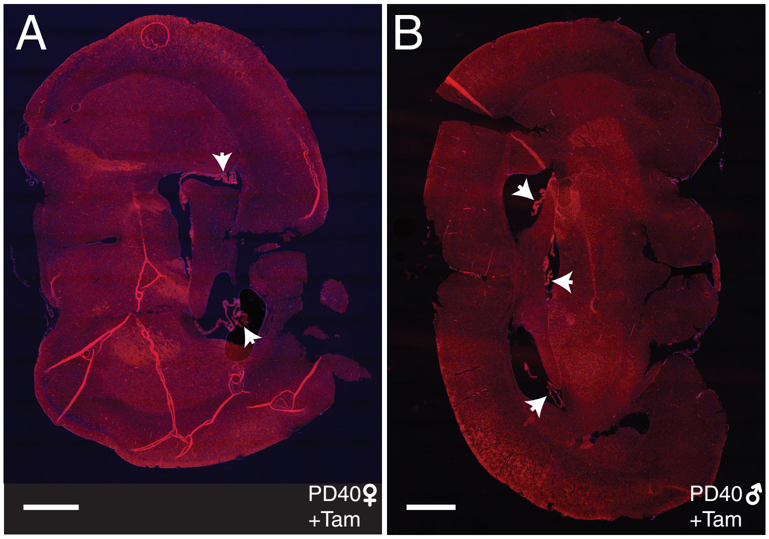

**Figure S5.** *Lack of Cre recombinase activity in $Rpe65^{CreERT2}$ mouse brain as assessed with the mT/mG reporter.* Panels **A** and **B** show whole brain cryo-sections from $Cre^{+/-}$ female (**A**) and male (**B**) mice following Cre induction with IP tamoxifen starting on PD21 (n = 2). Brains were processed 2 weeks after the last dose of tamoxifen. No green fluorescence was evident in the sections, including the choroidal plexus epithelium (white arrows) confirming the absence of Cre-mediated recombination in the brains of $Rpe65^{CreERT2}$ mice. The scale bar indicates 1000 μm.

# Figure S6

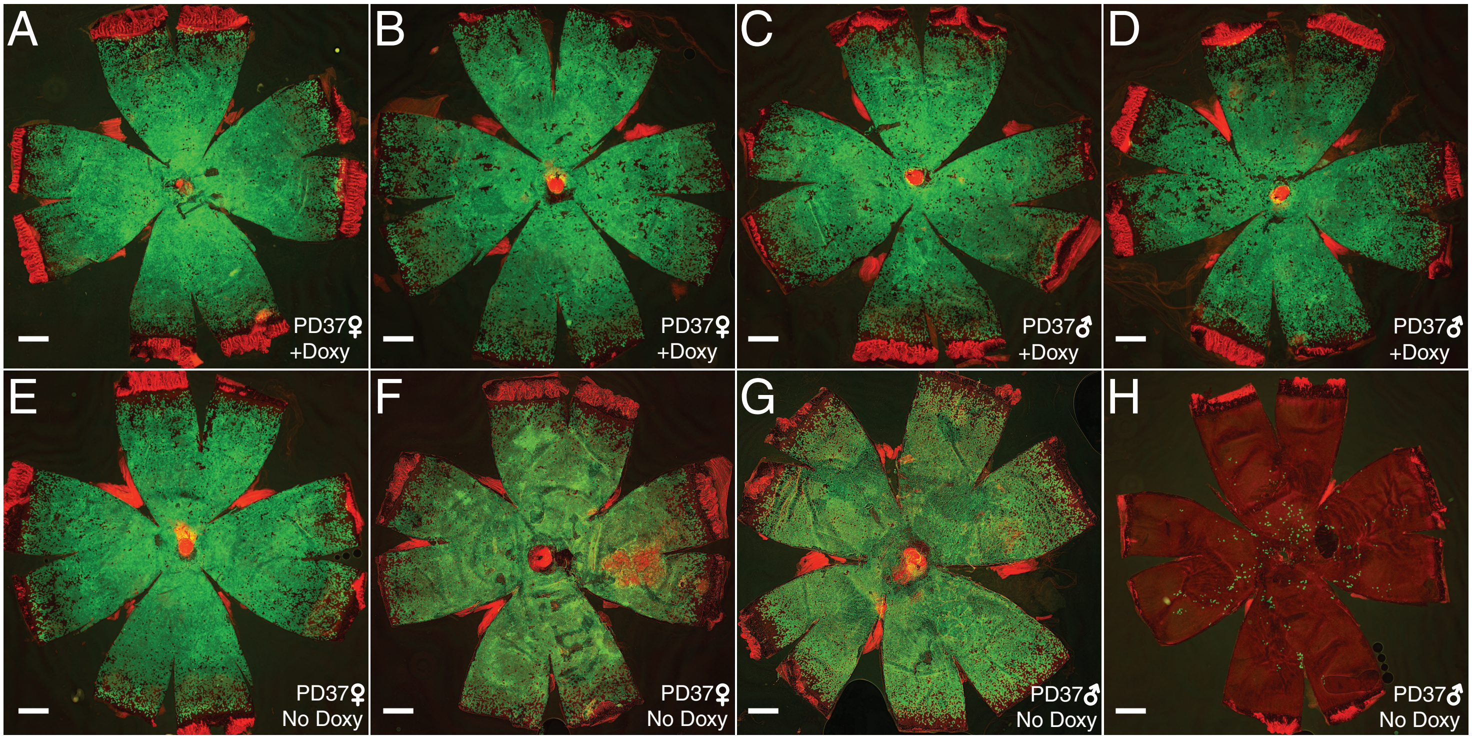
A
PD37♀
+Doxy
B
PD37♀
+Doxy
C
PD37♂
+Doxy
D
PD37♂
+Doxy
E
PD37♀
No Doxy
F
PD37♀
No Doxy
G
PD37♂
No Doxy
H
PD37♂
No Doxy

**Figure S6.** *Cre recombinase activity in tet-ON VMD2-Cre mouse retina as assessed with the mT/mG reporter.* Panels **A-D** show RPE flatmounts from representative *VMD2-Cre*$^{+/-}$ female (**A, B**) and *VMD2-Cre*$^{+/-}$ male (**C, D**) mice administered 8 mg of doxycycline (Doxy) in water by oral gavage for two consecutive days starting on PD21 (n = 4). The flatmounts were obtained two weeks after the final dose of doxycycline. Green fluorescence indicates cells where Cre-mediated recombination had occurred, whereas red fluorescence indicates un-recombined cells. Panels **E-H** show RPE flatmounts from representative *VMD2-Cre*$^{+/-}$ female (**E, F**) and *VMD2-Cre*$^{+/-}$ male (**G, H**) mice administered vehicle (water) by oral gavage for two consecutive days starting on PD21 (n = 4). The flatmounts were obtained two weeks after the final water gavage. Note the variability in doxycycline-independent induction in these mice. The scale bar indicates 500 μm.

# Figure S7

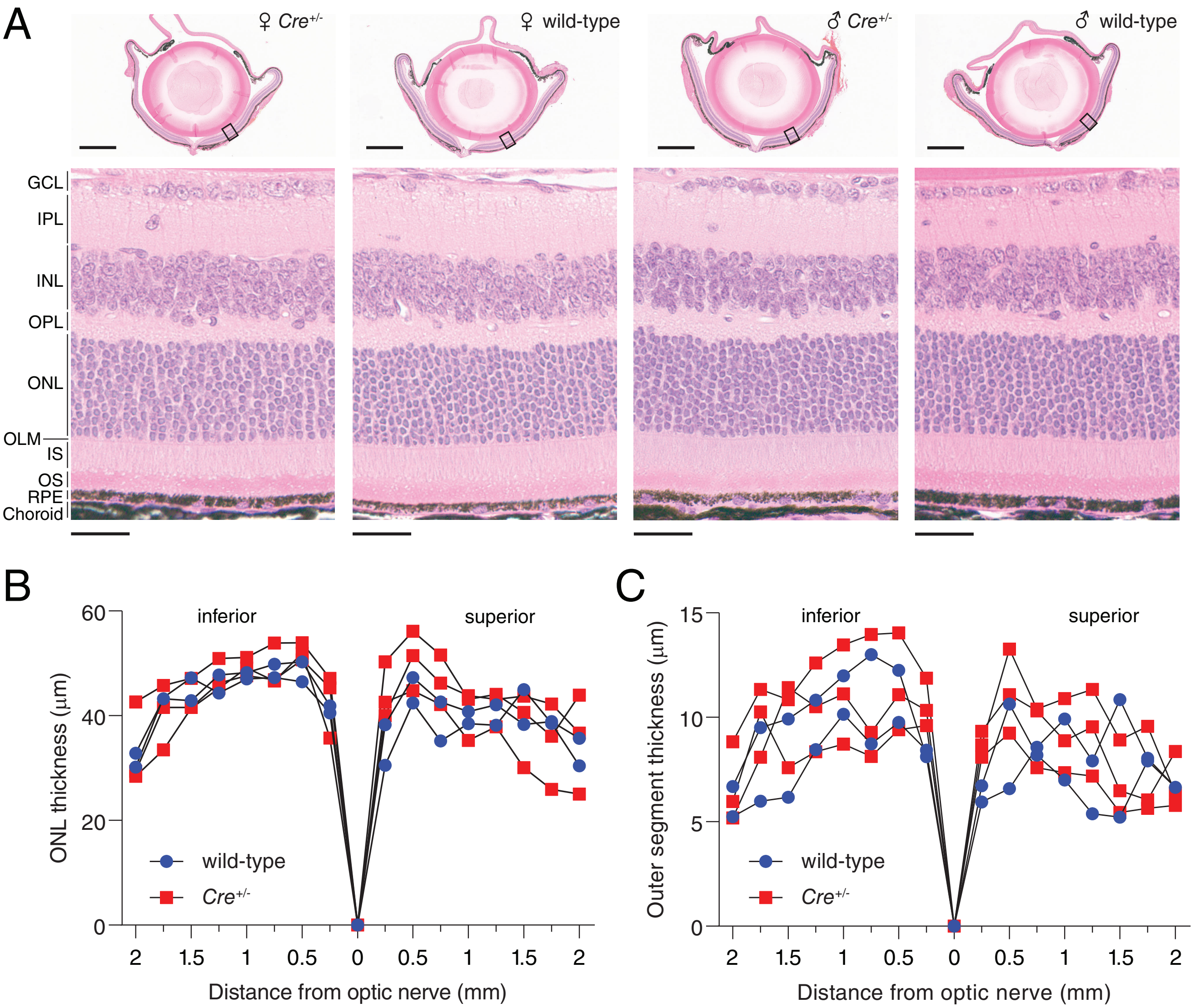

A
♀ Cre+/-
♀ wild-type
♂ Cre+/-
♂ wild-type
GCL
IPL
INL
OPL
ONL
OLM
IS
OS
RPE
Choroid
B
inferior
superior
ONL thickness (µm)
wild-type
Cre+/-
Distance from optic nerve (mm)
C
inferior
superior
Outer segment thickness (µm)
wild-type
Cre+/-
Distance from optic nerve (mm)

**Figure S7.** *Retinal histology of 2-month old, IP tamoxifen-treated $Cre^{+/-}$ mice and wild-type littermates.* **A**) Hematoxylin and eosin-stained retinal paraffin sections from $Cre^{+/-}$ mice (n = 3) and wild-type littermate controls (n = 2) that were treated with IP tamoxifen for five days starting on PD21. The upper panels show the complete retinal section. The lower panels show zoomed images corresponding to the areas boxed in black rectangles in the upper panels. The scale bars in the upper and lower panels indicate 600 μm and 25 μm, respectively. Abbreviations: GCL, ganglion cell layer; INL, inner nuclear layer; IPL, inner plexiform layer; IS, inner segment; OLM, outer limiting membrane; ONL, outer nuclear layer; OPL, outer plexiform layer; OS, outer segment; RPE, retinal pigment epithelium. **B**) Quantification of the outer nuclear layer (ONL) thickness as a function of distance from the optic nerve revealed comparable thickness between $Cre^{+/-}$ and wild-type mice. **C**) Quantification of the photoreceptor outer segment thickness as a function of distance from the optic nerve revealed comparable thickness between $Cre^{+/-}$ and wild-type mice.

**Table S1.** RPE-targeted Cre mouse lines reported to date.

| Name | Targeting strategy | Expression driver | Cre recombinase | Characteristics | Reference |
|---|---|---|---|---|---|
| *Mct3*-Cre | random transgene integration | Monocarboxylate transporter 3 (*Mct3*) promoter | iCreER$^{T2}$ | • Tamoxifen inducible<br>• Low and mosaic recombination | (2) |
| tet-ON VMD2-Cre | random transgene integration | Vitelliform macular dystrophy-2 (*Vmd2*), promoter | Cre | • Doxycycline inducible<br>• Inducer-independent Cre expression<br>• Mosaic recombination | (3) |
| *Tyr*-CreER$^{T2}$ | random transgene integration | Hsp70 promoter and Tyr enhancer | CreER$^{T2}$ | • Tamoxifen inducible<br>• Prenatal and post-natal induction possible<br>• Mosaic recombination<br>• Cre expression in ciliary body | (4) |
| *Mart1*-Cre | random transgene integration | Melanoma-Associated Antigen Recognized by T-cells (MART-1) promoter | Cre | • Constitutive Cre activity<br>• Recombination not specific to the RPE | (5) |
| *Trp1*-Cre | random transgene integration | Tyrosinase-related protein-1 (Trp1) promoter | Cre | • Constitutive Cre activity<br>• Mosaic recombination<br>• Recombination not specific to the RPE<br>• Intrinsic RPE toxicity | (6, 7) |
| *Dct*-Cre | random transgene integration | dopachrome tautomerase (Dct) promoter | Cre | • Constitutive Cre activity<br>• Recombination not specific to the RPE | (8) |
| *BEST1*-Cre | random transgene integration | Bestrophin-1 (Best1) promoter | Cre | • Constitutive Cre activity<br>• Mosaic recombination<br>• Intrinsic RPE toxicity | (9, 10) |
| *BEST1*-CreER$^{T2}$ | Targeted transgene integration at ROSA26 locus | Bestrophin-1 (Best1) promoter | CreER$^{T2}$ | • Mosaic recombination<br>• Cre activity in a population of Müller glia | (11) |

**Table S2.** PCR conditions used for animal genotyping

| Locus | Primers (5' – 3') | Cycling parameters | | Bands generated (bp) | Notes | Reference |
|---|---|---|---|---|---|---|
| | | Time | Temperature (°C) | | | |
| *Rpe65*$^{CreERT2}$ | **RPE65_E14f:** CTTCCATGGACTGTTC AAAAGATCC<br><br>**RPE65_E14r:** AACTTCCAGGAGTAAG TTCTGTCC<br><br>**RPE65_CreERT2:** GCATAACCAGTGAAAC AGCATTG | 5 min<br>45 sec<br>30 sec<br>1 min<br>10 min | 94<br>94 \|<br>63 \| x 35 cycles<br>72 \|<br>72 | 449 (*Cre*$^{+/+}$)<br><br>449 and 146 (*Cre*$^{+/-}$)<br><br>146 (wild-type) | | This work |
| mT/mG | **12177:** CTTTAAGCCTGCCCAG AAGA<br><br>**30297:** TAGAGCTTGCGGAACC CTTC<br><br>**30298:** AGGGAGCTGCAGTGG AGTAG | 5 min<br>45 sec<br>30 sec<br>1 min<br>10 min | 94<br>94 \|<br>60 \| x 35 cycles<br>72 \|<br>72 | 128 (mT/mG$^{+/+}$)<br><br>128 and 212 (mT/mG$^{+/-}$)<br><br>212 (wild-type) | | (12) |
| RPE65 (L/M 450 variation) | **RPE65f:** GCATACGGACTTGGGT TGAATCAC<br><br>**RPE65r:** GGTTGAGAAACAAAGA TGGGTTCAG | 5 min<br>45 sec<br>30 sec<br>1 min<br>10 min | 94<br>94 \|<br>60 \| x 35 cycles<br>72 \|<br>72 | 231 (M450)*<br><br>231, 142, 89 (M450/L450)*<br><br>142, 89 (L450)* | * Following digestion of the PCR product with MwoI. The L450 variant contains an MwoI site at nt 142–151 that is absent in the M450 variant. Digestion with MwoI therefore results in two bands of ~142 and 89 bp for L450, and one band of 231 bp for M450. | (13) |
| rd8 | **mCrb1-mF1:** GTGAAGACAGCTACAG TTCTGATC<br><br>**mCrb1-mF2:** GCCCCTGTTTGCATGG AGGAAACTTGGAAGAC AGCTACAGTTCTTCTG<br><br>**mCrb1-mR:** GCCCCATTTGCACACT GATGAC | 5 min<br>45 sec<br>30 sec<br>1 min<br>10 min | 94<br>94 \|<br>60 \| x 35 cycles<br>72 \|<br>72 | 220 (wild-type)*<br><br>244 (rd8)* | * The mF1 x mR (wild-type) and mF2 x mR (rd8) reactions are run separately because of the similarity in their product sizes. | (14) |
| VMD2-Cre | **VMD2-Cre_f:** GAACCTGATGGACATG TTCAG<br><br>**VMD2-Cre_r:** AGTGCGTTCGAACGCT AGAGC | 5 min<br>45 sec<br>30 sec<br>1 min<br>10 min | 94<br>94 \|<br>60 \| x 35 cycles<br>72 \|<br>72 | 375 (*Cre*$^{+}$)* | * The genotyping protocol does not distinguish between heterozygous and homozygous Cre animals | (15) |

## SUPPLEMENTAL REFERENCES


1. Huang J, Possin DE, and Saari JC. Localizations of visual cycle components in retinal pigment epithelium. *Mol Vis.* 2009;15:223-34.

2. Longbottom R, Fruttiger M, Douglas RH, Martinez-Barbera JP, Greenwood J, and Moss SE. Genetic ablation of retinal pigment epithelial cells reveals the adaptive response of the epithelium and impact on photoreceptors. *Proc Natl Acad Sci USA.* 2009;106(44):18728-33.

3. Le YZ, Zheng W, Rao PC, Zheng L, Anderson RE, Esumi N, et al. Inducible expression of cre recombinase in the retinal pigmented epithelium. *Invest Ophthalmol Vis Sci.* 2008;49(3):1248-53.

4. Schneider S, Hotaling N, Campos M, Patnaik SR, Bharti K, and May-Simera HL. Generation of an inducible RPE-specific Cre transgenic-mouse line. *PLoS ONE.* 2018;13(11):e0207222.

5. Aydin IT, and Beermann F. A MART-1::Cre Transgenic Line Induces Recombination in Melanocytes and Retinal Pigment Epithelium. *Genesis.* 2011;49(5):403-9.

6. Mori M, Metzger D, Garnier JM, Chambon P, and Mark M. Site-specific somatic mutagenesis in the retinal pigment epithelium. *Invest Ophthalmol Vis Sci.* 2002;43(5):1384-8.

7. Thanos A, Morizane Y, Murakami Y, Giani A, Mantopoulos D, Kayama M, et al. Evidence for baseline retinal pigment epithelium pathology in the Trp1-Cre mouse. *Am J Pathol.* 2012;180(5):1917-27.

8. Guyonneau L, Rossier A, Richard C, Hummler E, and Beermann F. Expression of Cre recombinase in pigment cells. *Pigment Cell Res.* 2002;15(4):305-9.

9. Iacovelli J, Zhao C, Wolkow N, Veldman P, Gollomp K, Ojha P, et al. Generation of Cre transgenic mice with postnatal RPE-specific ocular expression. *Invest Ophthalmol Vis Sci.* 2011;52(3):1378-83.

10. He L, Marioutina M, Dunaief JL, and Marneros AG. Age- and gene-dosage-dependent cre-induced abnormalities in the retinal pigment epithelium. *Am J Pathol.* 2014;184(6):1660-7.

11. Chen M, Kim L, Lu CW, Zeng H, and Vollrath D. An efficient inducible RPE-Selective cre transgenic mouse line. *Exp Eye Res.* 2020:108370.

12. Muzumdar MD, Tasic B, Miyamichi K, Li L, and Luo LQ. A global double-fluorescent cre reporter mouse. *Genesis.* 2007;45(9):593-605.

13. Lopes VS, Gibbs D, Libby RT, Aleman TS, Welch DL, Lillo C, et al. The Usher 1B protein, MYO7A, is required for normal localization and function of the visual retinoid cycle enzyme, RPE65. *Hum Mol Genet.* 2011;20(13):2560-70.

14. Mattapallil MJ, Wawrousek EF, Chan CC, Zhao H, Roychoudhury J, Ferguson TA, et al. The Rd8 mutation of the Crb1 gene is present in vendor lines of C57BL/6N mice and embryonic stem cells, and confounds ocular induced mutant phenotypes. *Invest Ophthalmol Vis Sci.* 2012;53(6):2921-7.

15. Chaix A, Lin T, Le HD, Chang MW, and Panda S. Time-Restricted Feeding Prevents Obesity and Metabolic Syndrome in Mice Lacking a Circadian Clock. *Cell Metab.* 2019;29(2):303-19 e4.
